# High‐Performance MXene Hydrogel for Self‐Propelled Marangoni Swimmers and Water‐Enabled Electricity Generator

**DOI:** 10.1002/advs.202408161

**Published:** 2024-11-18

**Authors:** Jiayi Zhou, Yan Zhang, Ming Zhang, Dongye Yang, Wenwei Huang, Ao Zheng, Lingyan Cao

**Affiliations:** ^1^ School of Material Science and Engineering Shanghai University of Engineering Science Shanghai 201620 P. R. China; ^2^ Department of Prosthodontics Shanghai Ninth People's Hospital Shanghai Jiao Tong University School of Medicine Shanghai 200011 P. R. China; ^3^ College of Stomatology Shanghai Jiao Tong University Shanghai 200011 P. R. China

**Keywords:** hydrogels, nanoconfined channels, self‐power, self‐propel, water‐enabled electricity generators

## Abstract

Developing multifunctional materials that integrate self‐propulsion and self‐power generation is a significant challenge. This study introduces a high‐performance MXene‐chitosan composite hydrogel (CM) that successfully combines these functionalities. Utilizing Schiff base bond and hydrogen bond interactions, the CM hydrogel, composed of chitosan, vanillin, and MXene, achieves exceptional self‐propulsion on water driven by Marangoni forces. The hydrogel demonstrates rapid movement, extended operation, and controllable trajectories. Notably, the CM hydrogel also exhibits superior degradability, recyclability, and repeatability. Furthermore, the nano‐confined channels within the hydrogel play a crucial role in enhancing its water‐enabled electricity generation (WEG) performance. By efficiently adsorbing water molecules and selectively transporting cations through these channels, the hydrogel can generate electricity from water molecules and cations more efficiently. As a result, the CM‐WEG achieves a stable open‐circuit voltage of up to 0.83 V and a short‐circuit current of 0.107 mA on seawater, with further improvements in K_2_CO_3_‐containing water, reaching 1.26 V and 0.922 mA. Leveraging its unique combination of self‐propulsion and WEG functionalities, the CM hydrogel is successfully used for cargo delivery while simultaneously powering electronic devices. This research represents a significant step toward the development of self‐powered, autonomous soft robotics, opening new research directions in the field.

## Graphical abstract

This study presents a high‐performance hydrogel (CM) that seamlessly integrates self‐propulsion and self‐power generation functionalities through a nanoconfined structure. The hydrogel demonstrates exceptional locomotor capabilities on the water surface and efficient water‐enabled electricity generation. Furthermore, the potential application of this hydrogel in cargo delivery, coupled with the simultaneous electronic devices powering, is discussed, thereby paving the way for advancements in self‐powered soft robotics.

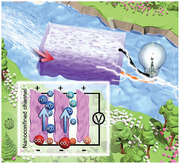

## 1 Introduction

Autonomous self‐propelled soft robots that can move and navigate independently of external power sources are attracting increasing attention.^[^ ^1^ ^]^ Various propulsion methods, such as magnetic,^[^ ^2^ ^]^ optical,^[^ ^3^ ^]^ and chemical^[^ ^4^ ^]^ drives have been developed for swimming locomotion. Although methods based on external stimuli demonstrate considerable motion controllability, the cumbersome nature of external stimuli sources restricts the practical application of these soft robots.^[^ ^5^ ^]^ Natural semiaquatic insects, notably Stenus^[^ ^6^ ^]^ and Velia,^[^ ^7^ ^]^ can traverse water surfaces by utilizing the Marangoni effect. This effect is realized by creating a gradient in the surface tension,^[^ ^8^ ^]^ which can result from the use of surfactants or temperature differences.^[^ ^9^ ^]^ Fluid flow induced through this surface tension gradient enables the robot to self‐propel using the Marangoni effect,^[^ ^10^ ^]^ eliminating the need for an external energy source^[^ ^11^ ^]^ or control mechanisms.^[^ ^12^ ^]^ For soft robots, Wu et al.^[^ ^9b^ ^]^ proposed a motion control strategy that relies on the surface tension gradients generated by surfactants. Zhao et al.^[^ ^13^ ^]^ fabricated an aligned hollow fiber motor that self‐propelled along a water surface based on the Marangoni effect using ethanol as the fuel to generate a surface tension gradient. While significant progress has been made in the domain of self‐propelled robots based on the Marangoni effect, certain limitations and challenges remain and warrant further investigation.

Marangoni‐driven hydrogels are typically considered to require the addition of an extra component (usually a surfactant or an organic solvent) implemented in the reaction to create the surface tension gradient, impacting the efficiency of the hydrogel's self‐propelling performance.^[^ ^14^ ^]^ When fuel is directly added to the interface, additional transport mechanisms and storage of large amount of fuel are needed,^[^ ^15^ ^]^ which compromises autonomy and efficiency, and hinders miniaturization.^[^ ^16^ ^]^ The encapsulation of fuel by absorbent matrices (hydrogels^[^ ^17^ ^]^ and metal‐organic frameworks^[^ ^18^ ^]^) results in weak interactions between the matrices and fuel, leading to low motion efficiency, a short lifespan, and uncontrolled motion trajectories.

Apart from inducing motion through a concentration gradient, Mei et al.^[^ ^1b^ ^]^ observed the locomotion of water striders and synthesized a new type of self‐propelled hydrogel with dynamic hydrophobic properties. Water‐surface robots, which create asymmetric surface tension through dynamic wetting processes, encounter significant challenges in regard to locomotion due to their potential attraction and adherence to hydrophobic boundaries.^[^ ^19^ ^]^ Despite significant advancements in biomimetic robot materials, research on self‐propelling soft materials remains in its early stages. The development of methods for efficient motion by mimicking the functions of aquatic organisms is a challenging task.

Currently, most self‐propelled robots rely on external power sources to operate additional devices or perform complex tasks, such as running sensors and robotic arms.^[^ ^20^ ^]^ This dependence poses a significant challenge to achieving autonomous electric power generation. Water, as a clean, abundant, and renewable energy source,^[^ ^21^ ^]^ has tremendous potential for electricity generation.^[^ ^22^ ^]^ Water‐enabled electricity generation (WEG) technologies leverage the interactions between water molecules and hygroscopic materials to produce stable and long‐term electricity.^[^ ^23^ ^]^ The performance of WEG is profoundly influenced by the surface charge density and channel size of the materials.^[^ ^24^ ^]^ Hydrogels, which are composed of polymer chains with hydrophilic groups, can spontaneously absorb and transmit water molecules as electric energy;^[^ ^25^ ^]^ this makes them an attractive option for energy harvesting, offering the potential for self‐powered and sustainable energy sources across various applications, such as powering sensors,^[^ ^26^ ^]^ actuating devices,^[^ ^27^ ^]^ and even charging batteries.^[^ ^28^ ^]^ Despite significant advances, the power density and conversion efficiency reported to date are still too low for practical applications.

Herein, we propose a novel, degradable, and recyclable hydrogel that exhibits remarkable self‐propulsion and water‐electric generation capabilities, offering high repeatability. The hydrogel is composed of Schiff‐based bonds and hydrogen bonds, enabling self‐motion through the breaking of Schiff‐based bonds and the release of vanillin, which in turn creates a concentration gradient and dynamic wetting processes. The operational principle of these hydrogel robots can be validated through computational fluid dynamics simulations, allowing for precise design of motion trajectories by controlling the shape of the hydrogel and its installation site. Furthermore, the hydrogel's surface functional groups and micro/nano channels significantly enhance its water adsorption capacity and facilitate selective ion migration via nanoconfinement effects, ultimately leading to an increased generation of electrical energy. Connecting multiple devices in a linear manner allows for the amplification of voltage and current to facilitate the operation of power electronics. Finally, we provided a proof‐of‐concept for the unique potential applications of the hydrogel in specific water‐related tasks, such as cargo delivery and ship power sources. These findings provide promising opportunities for practical applications of hydrogel‐based energy generation and self‐propelling robots.

## 2 Results and Discussion

### 2.1 Fabrication and Characterization of Hydrogel Robots

**Figure 1:**
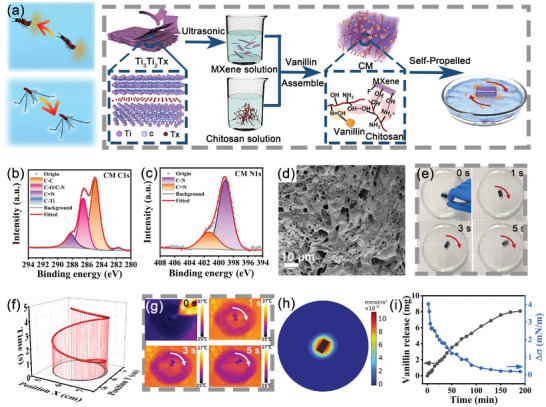
Fabrication and characterization of hydrogel robots. a) A schematic depiction of the CM hydrogel robot preparation process, illustrating locomotive behavior reminiscent of water striders and Stenus. The b) C 1s and c) N 1s XPS spectra of the CM hydrogel. d) SEM image of the CM hydrogel. e) Movement snapshots and f) trajectory analysis of the rectangular CM hydrogel. g) Observations of the concentration gradient across the water surface captured with an infrared imaging camera. h) A simulation depicting the concentration of vanillin in the vicinity of the CM hydrogel robot when it was placed in water. i) Monitoring of vanillin release and the consequent surface tension gradient changes in the CM hydrogel.

We synthesized self‐propelled hydrogel robots inspired by the motion behavior of water striders and Stenus (**Figure** **1**a). X‐ray photoelectron spectroscopy (XPS) confirmed the formation of the MXene‐chitosan composite hydrogel (CM), characterized by dynamic Schiff base bonds between chitosan and vanillin (Figure 1b,c). The Fourier transform infrared (FTIR) spectrum of CM shows a new peak at 1637 cm⁻¹, corresponding to C═N stretching, which was absent in the spectra of chitosan and vanillin (Figure S1, Supporting Information), confirming the presence of Schiff base bonds in the composite.^[^ ^29^ ^]^ Additionally, compared to that of the CS hydrogel, the C─OH stretching peak at 3422 cm⁻¹ shifts to a lower wavenumber with MXene incorporation, indicating the increased density of hydrogen bonding. These analyses demonstrate that CM forms through dynamic Schiff base bonds between chitosan and vanillin and multiple hydrogen bonds involving MXene, chitosan, and vanillin. The CM hydrogel exhibited an interpenetrating 3D network structure (Figure 1d).

When placed on the water surface, the rectangular hydrogel rotates rapidly, with the rotation driven by the highest torque generated due to its geometric symmetry (Figure 1e; Video S1, Supporting Information). To visually depict the movement of hydrogel robots, we extracted their displacements at various times from the video (Figure 1f). The hydrogel robots displayed both revolution (orbital motion) and spinning motion around their center, with a spinning period of 360°/s (Figure S2, Supporting Information). In addition, we conducted further analysis of the changes in the instantaneous velocity and frequency of velocity using a fast Fourier transform (Figure S3, Supporting Information). The initial average velocity of the hydrogel robot was measured at 1.83 cm s^−1^. Over the next 50 min, the velocity gradually decreased to 0 cm s^−1^, with occasional sudden increases in the instantaneous velocity peaking at 19.6 cm s^−1^. The decrease in the Schiff base bond content and increased pore size after movement confirmed that the Schiff base underwent partial fragmentation during the process (Figures S4–S6, Supporting Information).

When the CM robot was placed on the water surface, vanillin was released from the hydrogel through reversible and unstable Schiff‐based bonds. This process facilitates the diffusion of vanillin into the surrounding water, resulting in a temperature increase due to the release of heat. An infrared (IR) video camera was utilized to observe both the movement of the CM robot and the migration of vanillin molecules. As shown in Figure 1g and Video S2 (Supporting Information), the continuous rotation of the hydrogel resulted in the formation of a windmill‐shaped red zone with a high concentration of vanillin. The finite element analysis (FEA) was further employed to simulate the distribution of vanillin molecules in solution upon their release from the CM robots (Figure 1h). Initially, asymmetric diffusion occurs upon the placement of the robot on the water surface, leading to the appearance of distinct regions with different concentrations of vanillin. The increased concentration of vanillin in water reduces the surface tension (Figure 1i; Figure S7, Supporting Information). The asymmetric diffusion creates areas with both low and high surface energy, consequently generating Marangoni pressure.^[^ ^13^ ^]^ Due to the increased temperature of the released vanillin, we further calculated a temperature gradient and conducted surface tension simulations. It was confirmed that the surface tension induced by the temperature gradient has a minimal effect on the self‐propulsion of the robot (Figure S8, Supporting Information). Thus, the CM robot contributes to the formation of a concentration gradient as vanillin diffuses from the hydrogel to the water surface. This gradient induces the generation of Marangoni flow, inducing the rotation of the hydrogel robot on the water surface.

### 2.2 Motion Capability of Hydrogel Robots and Mechanisms of Locomotion

To verify the universality of the movement model of the hydrogels based on Schiff‐based bonds, we examined the impact of hydrogel structure on the autonomous locomotion rate and duration. Chitosan hydrogel (CS), chitosan‐graphene oxide hydrogel (CG), and CM prepared through the freeze‐thawing method (FTCM) were also crosslinked using Schiff‐based bonds and hydrogen bonds to form a 3D porous structure (Figure S9, Supporting Information). IR and XPS results (Figure 1b,c; Figures S1, S10 and S11, Supporting Information) indicate that the hydrogen bond content followed the order CG > FTCM > CM > CS. Additionally, the Schiff‐based bond content was ranked as follows: CM > CS > CG > FTCM. The CG hydrogel exhibited the highest number of hydrogel bonds due to the abundant oxygen‐containing functional groups on its surface. In the case of FTCM, the chitosan chain undergoes rearrangement during the freeze‐thaw cycles, leading to the hydrolysis of the Schiff‐based bond by water molecules and a decrease in the Schiff‐based bond content.

**Figure 2:**
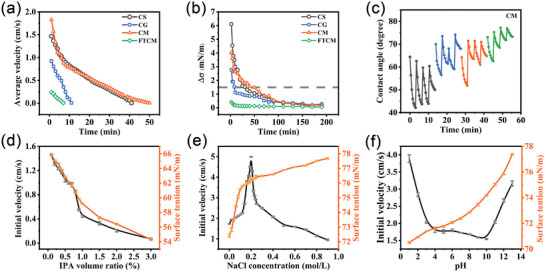
Motion capabilities and locomotion mechanisms of hydrogel robots. Changes in the a) average velocity and b) surface tension gradient of the CS, CG, CM, and FTCM hydrogel robots. c) Changes in the contact angle of the CM hydrogels. The initial velocities of the CM hydrogel when it was traveling through solutions with diverse surface tension coefficients: d) an IPA aqueous solution, featuring a lower surface tension than water, and e) a NaCl solution, with a higher surface tension than water, and f) solutions with increased pH values corresponding to increased surface tension.

All hydrogels demonstrated autonomous movement (**Figure** **2**a). The CM robot initially showed a velocity of 1.83 cm s^−1^, gradually decreasing to 0 cm s^−1^ after 50 min. The CS robot displayed an initial velocity of 1.47 cm s^−1^ and can sustain movement for 41 min. The surface tension gradient (△δ, Figure 2b) is closely related to the released vanillin content, which, in turn, is associated with Schiff‐based bond content (Figure S12, Supporting Information). Hydrogels with high amounts of Schiff‐based bonds, such as CS and CM, exhibit higher initial velocities and longer locomotion durations. By contrast, the CG hydrogel had more hydrogen bonds and fewer Schiff‐based bonds, resulting in a lower initial velocity of 0.92 cm s^−1^ and a locomotion of 11 min. When △δ decreased to ≈1.5 mN m^−1^, the Marangoni force was insufficient to induce the motion of the CG robot. The release of vanillin creates a surface tension gradient, generating the Marangoni force, which serves as the main driving force for the locomotion of the hydrogel. Interestingly, FTCM released a small amount of vanillin, with a △δ much smaller than 0.5 mN m^−1^; however, it can still continue to move for 7 min. Although the CS robot initially released a larger amount of vanillin than the CM robot, the CM robot exhibited a higher initial velocity.

To better understand the propulsion mechanism of autonomous movement, we conducted dynamic wetting process measurements. In Figure 2c, the contact angle of the hydrogel during water absorption was measured using the sessile droplet technique.^[^ ^1b^ ^]^ The initial contact angle of the CM was 64°, and gradually decreased to 42° during the first 3 min as the hydrogel absorbed water from the droplets. When a droplet was added to the adjacent position of the hydrogel (red line), the contact angle increased to 70°. This change in the contact angle is attributed to the migration of hydrophilic functional groups during water absorption, which causes the unwetted part of the hydrogel to expose nonpolar parts of the backbone to the air. The dynamic wetting process gives rise to an uneven surface tension distribution around the hydrogel, resulting in its movement toward the regions of higher surface tension through Marangoni propulsive forces. Compared with other materials, the CM hydrogel exhibited significant dynamic wetting, generating strong Marangoni propulsive forces and enhancing its lifetime and efficiency (Figure 2c; Figure S13, Supporting Information). Even though the CS hydrogel exhibits a larger surface tension gradient due to the concentration gradient (Figure 2b), its lower rate of change in dynamic contact angle results in a lower initial velocity compared with the CM hydrogel (Figure 2a; Figures S13 and S14, Supporting Information). By contrast, the CG hydrogel, which contains a high amount of hydrogen bonds and shows strong structural stability, did not experience dynamic wetting (Figure S14b, Supporting Information). Although FTCMs could not generate sufficient Marangoni propulsive forces due to the vanillin concentration gradient, the resulting hydrogels exhibit dynamic wetting (Figure S14c, Supporting Information), enabling them to move on water surfaces for a short period of time. Thus, various active Schiff‐based hydrogels could autonomously move in aqueous solutions due to Marangoni propulsive forces, demonstrating the universality of the synthetic self‐propelled hydrogel method.

The effect of surface tension gradients on the motion capability was further explored by adjusting the surface tension of the solution by NaCl and isopropyl alcohol (IPA) addition. As the concentration of IPA increases, the surface tension of the solution decreases, leading to a decrease in the initial speed of the CM robot (Figure 2d). However, increasing the surface tension by increasing the NaCl concentration leads to an initial velocity increase until the NaCl concentration reaches 0.2 mol L^−1^ (Figure 2e). A further increase in the NaCl concentration increases the solution viscosity,^[^ ^30^ ^]^ resulting in greater motion resistance and a decrease in the velocity (Figure 2e).

Under acidic conditions, the dynamic Schiff‐based reaction is more prone to hydrolysis,^[^ ^31^ ^]^ causing the structure to loosen and the porosity to increase (Figure S15a, Supporting Information). Although the initial surface tension shows a slight decrease in acidic conditions, the rapid release of vanillin gives rise to a higher surface tension gradient, leading to an increase in the initial rate (Figure 2f). Under alkaline conditions, protonated chitosan chains can rapidly neutralize and form a 3D network, helping maintain the pore structure and restricting the release of vanillin, which results in a slight decrease in the surface tension gradient (Figure S15b, Supporting Information). With increased pH values, the surface tension rises, causing the initial velocity to increase together with the increasing surface tension gradient (Figure 2f). Therefore, hydrogels with Schiff‐based bonds can exhibit locomotion in various aqueous solutions, and the velocity can be controlled by adjusting the pH and surface tension of the solution.

### 2.3 Locomotion Trajectory Control

**Figure 3:**
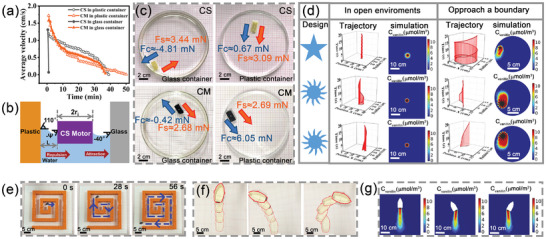
Control and simulation of locomotion trajectories. a) Changes in the average velocities of CS and CM hydrogel robots enclosed in glass or plastic containers. b) A cross‐sectional illustration of a CS hydrogel at the interface between air and water, emphasizing meniscus formation adjacent to polystyrene (PS) and glass walls. c) Surface tension and transverse capillary forces are exerted on the CS and CM hydrogel robots contained within glass or PS containers. d) Design considerations for hydrogel robots. Locomotion of star‐ and gear‐shaped CM hydrogel robots in open environments and their approach to boundaries, complemented by simulations of dynamic vanillin concentration changes using FEA. e) Navigation of CM hydrogel robots through a labyrinthine‐like structure. f) Regulation of the swimmer's trajectory through strategic placement of the hydrogel components and g) estimation of motion trajectories simulated using FEA.

The locomotion of hydrogel robots is significantly influenced by their surroundings. Specifically, within circular containers with a diameter of 14 cm, the rectangular CS and CM hydrogel robots (1 × 2 cm^2^) showed different locomotion properties when approaching a boundary (**Figure** **3**a). In a plastic container, both CM and CS robots demonstrated either revolution or orbital motion accompanied by spinning motions lasting ≈50 and 39 min, respectively. Conversely, in a glass container, the CM robot exhibited a spinning motion for at least 37 min, while the CS robot only moved for 76 s.

We hypothesize that the movement of hydrogel robots near a boundary is dictated by two key factors: capillary force (Fc) and surface tension (Fs), which are created by the concentration gradient. Figure 3b illustrates how the liquid surface surrounding an object forms a meniscus due to gravity. Upon approaching a boundary, the deformation of the meniscus gives rise to a horizontal capillary force. As depicted in Figure 3c, the Marangoni force drove the CS robot toward the wall when it was placed in the center of the container. Applying the Laplace formula,^[^ ^32^ ^]^ we calculated Fc to be −4.81 mN and Fs to be ≈3.44 mN when the CS robot was at in close proximity to the glass boundary (see additional information in Supporting Information for detailed calculations). Consequently, the CS robot was attracted to the glass boundary and adhered to it. Despite its a significant surface tension gradient, the CS robot only moved for 76 s, indicating that Fc counteracted the driving force generated by the surface tension gradient.

Near the glass boundary, the calculated Fc for the CM robot was a mere −0.42 mN, which was much smaller than the Fs (2.68 mN). As a result, the CM robot failed to be attracted to the glass boundary. When drawn near a plastic surface, both the CS and CM robots were repelled by the plastic surface; this can be attributed to the surface energy and surface tension of the plastic surface, which were lower than those to the glass surface. The calculated Fc for both robots was larger than 0. The synergistic effect of the capillary force and surface tension drives the CS and CM robots to move away from the plastic boundary, thereby resulting in significantly longer locomotion durations. Hence, the movement of hydrogel robots is influenced by the capillary force between the robot and the boundary, necessitating a delicate equilibrium between capillary force and surface tension. An enhanced understanding of these interactions may open new directions for achieving more efficient and controlled movement of hydrogels in various environments.

Control of locomotion trajectory is essential in the development of self‐propelled devices. Insights into the influence of geometry on the locomotion of hydrogel robots can facilitate their design optimization for particular environments and applications. In our experiments, we discerned that a square CM robot with a degree of symmetry exhibited a circular motion trajectory (Figure S16, Supporting Information). To assess the impact of the aspect ratio (L/W) on the motion characteristics, we compared the initial velocities and motion duration of CM robots with varying aspect ratios and the same width. Our findings indicate that with an increase in the aspect ratio from 1 to 2.5, the initial velocity decreased from 2.41 to 0.79 cm s^−1^, and the duration increased from 15 to 49 min (Figure S17, Supporting Information). This phenomenon can be attributed to the decrease in the specific surface area resulting from an increase in the L/W aspect ratio, which decreased the Schiff‐based bond hydrolysis rate. Consequently, this approach significantly decreased the initial velocity and lengthened the duration of motion of the CM robot. Moreover, we observed that the circular CM robot demonstrated both translational and rotational motion during its operation (Figure S18, Supporting Information). Circles with smaller diameters experienced increased torque due to interface instability, consequently enhancing their likelihood of rapid rotation. We also noted an increase in the spin ratio corresponding to the reduction in the motor diameter.

The star‐shaped and gear CM robots initiate rotational movements about their axes (Figure 3d; Video S3, Supporting Information). The star‐shaped and gear CM robots exhibit rotational movements about their axes. In open environments, the motion of star‐shaped and gear CM robots is determined solely by their shapes. The direction of rotation depended on the relative arrangement of the curved and straight teeth of the gear. Upon positioning the curved teeth clockwise in relation to the straight teeth, the gear robot correspondingly rotated clockwise, and vice versa. FEA was utilized to simulate dynamic changes in the concentration of vanillin. A greater amount of vanillin was released from the curved teeth of the gears than the straight teeth. This leads to decreased surface tension at the curved teeth, thereby inducing the gear to rotate toward these areas. FEA was employed to simulate the locomotion trajectory of star‐shaped and gear CM robots as they approach a boundary. At the apex of the star‐shaped robot, an asymmetric vanillin concentration gradient forms adjacent to the wall, leading to asymmetric diffusion. Influenced by osmotic pressure, vanillin diverges, with the divergence angle undergoing periodic changes, facilitating lateral movement along the wall. This capability allows the robot to autonomously navigate topographical changes and achieve directed motion, without the need for external energy guidance (Video S4, Supporting Information). The experimental results corroborate finite element simulation predictions, demonstrating a tendency of the robots to move along walls by spinning. Generally, the geometric characteristics strongly influence the locomotive behavior of hydrogels. The geometric form of the hydrogel impacts the Marangoni propulsive force through its effect on the dispersion of vanillin within the solution. Understanding the impact of geometric forms on robot dynamics can aid in the process of design and fine‐tuning of the robots for target applications and environmental conditions.

We further investigated the capability of the robot to maneuver and extricate itself from constricted tortuous pathways, inclusive of maze‐like structures. The engineered CM robot employed a concentration‐mediated Marangoni force for forward propulsion. Upon encountering boundaries within constricted tortuous pathways, the robot's trajectory is modulated by capillary forces. Consequently, the robot successfully navigated its way out of a labyrinthine structure (Figure 3e; Video S5, Supporting Information).

Additionally, hydrogels can serve as driver modules, regulating the swimmers’ motion trajectory by manipulating the positioning of hydrogels. Three distinct configurations involving the placement of hydrogels in various sections of the stern were investigated. As illustrated in Figure 3f,g and Video S6 (Supporting Information), the vessels exhibited a range of trajectories, including straight lines, left‐turning courses, and right‐turning courses. The motion path of the Marangoni vessel can be forecasted through the calculation of the dispersive pattern of vanillin release, utilizing FEA. As a result, the preferred motion path of a vessel equipped with a hydrogel driving module can be deliberately designed and implemented.

### 2.4 Repeatability, Degradation, and Recyclability

**Figure 4:**
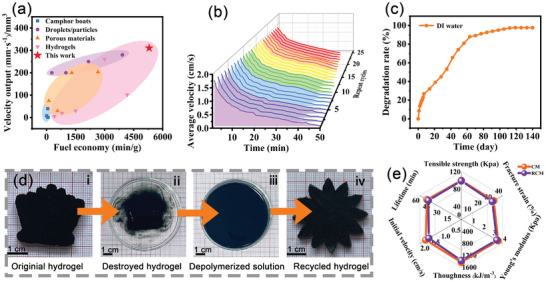
Repeatability, degradation, recyclability, and application. a) Self‐propelled motor benchmark. Significant factors for comparison of the performance of self‐propelled motors include the velocity output (maximum instantaneous velocity speed per unit volume) and fuel economy (maximum lifetime per unit mass). b) Average velocity of the CM hydrogel motor after 25 cycles. c) The degradation rate of the CM hydrogel within 140 days. d) Photographs of the recycling process. i) The original hydrogel, ii) the destroyed hydrogel, iii) the depolymerized solution, and iv) the recycled hydrogel. e) Performance of CM and RCM hydrogels.

In real‐world applications, paramount consideration must be given to fuel efficiency and velocity output, owing to their substantial influence on the operational expenses of a robotic system or vessel and their relevance to future mobility solutions. The CM robot demonstrates superior fuel efficiency and velocity output relative to earlier studies focusing on the systems utilizing the Marangoni effect (**Figure** **4**a; Table S1, Supporting Information). However, a fundamental constraint is imposed by the cessation of robot movement upon exhausting its fuel reserves while traversing the water‐air interface. This limitation can be circumvented in CM robots, because they can receive fuel augmentation via Schiff‐based reactions, thereby facilitating their reusability. Following immersion in a 5 wt.% vanillin solution for 1 h, the Schiff‐based bonds undergo regeneration, enabling the resumption of robot operation. The locomotive capability of the CM robot remains substantially unchanged, even after it was reused 25 times (Figure 4b)

Growing concerns regarding marine debris pollution have led to increased emphasis on controllable degradable materials for environmentally sustainable applications. In comparison to CS, a CM hydrogel exhibits improved stability, which can be attributed to the formation of more hydrogen bonds by MXene. The CM hydrogel exhibited observable stability over the initial 7 days of immersion in water, with a degradation rate of 26.84% (Figure 4c; Figure S19, Supporting Information). After 98 days, the degradation rate increased to 95.29% due to the disintegration of the hydrogel structure initiated by hydrolysis. Acidic conditions accelerate the cleavage of Schiff‐based bonds and the protonation of chitosan chains, leading to an increased degradation rate of the CM hydrogel. At pH 1.0, the degradation rate may reach 95.29% within just 7 h.

The singular geometric configuration of the CM robot might prove inadequate when faced with complex tasks or when multiple functionalities are needed across diverse contexts. Nevertheless, upon task completion, the CM robot can be recycled and repurposed for alternate applications (Figure 4d). To mitigate potential environmental pollution caused by hydrogels in marine environments, several feasible and effective strategies can be used for their recycling and repurposing. The original CM can be entirely decomposed into fragmented oligomers, which can subsequently undergo repolymerization with supplementary vanillin to formulate new CM hydrogel motors. This attribute endows the CM motor with superior recuperative capabilities, and the recycled CM hydrogel (RCM) demonstrates favorable mechanical properties and locomotion ability comparable to those of the original CM robot (Figure 4e).

### 2.5 Water‐Enabled Electricity Generation

**Figure 5:**
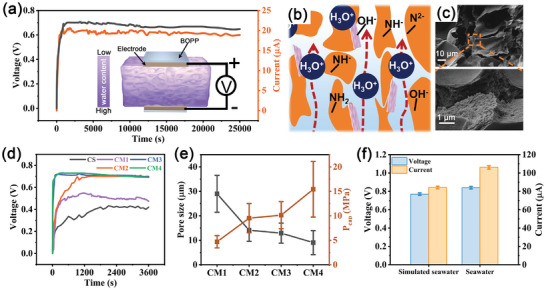
Water‐enabled electricity generation of the CM hydrogel‐based generator. a) Continuous V_OC_ output and short‐circuit current obtained by placing the CM‐WEG on water. b) Schematic of CM hydrogel water‐enabled electricity generation. c) The SEM image of the CM hydrogel. d) The V_OC_ of different hydrogels. e) Pore size and theoretical capillary pressure of CM1‐CM4 hydrogels, respectively. f) The V_OC_ of CM‐WEG in simulated seawater and actual seawater.

The CM‐WEG, fabricated using a CM hydrogel with Cu electrodes and encapsulated with commercial biaxially oriented polypropylene (BOPP) tape as shown in the inset of **Figure** **5**a, demonstrates exceptional capability for generating electrical energy from water. Compared to Pt electrodes, the CM‐WEG with Cu electrodes produces a larger stable open‐circuit voltage (VOC) of 0.7 V and a higher short‐circuit current (Isc) of 20.5 µA when placed on the water surface, due to the high conductivity of Cu electrodes (Figure 5a; Figure S20, Supporting Information). As previously reported, water molecules could be ionized by hydroxyl groups to produce free‐moving H_3_O^+^.^[^ ^33^ ^]^ The CM‐WEG has a large surface area and abundant functional groups to maximize water molecule adsorption, enhancing ion release at the hydrogel‐water interface (Figure 5b). Despite the higher hydroxyl content of the CG‐WEG, the superior performance of the CM‐WEG suggests that Schiff bases in the CM facilitate vanillin release (Figure S21, Supporting Information), with amino groups serving as adsorption sites for water molecules. The continuous hydrolysis of Schiff bases within the hydrogel facilitates the generation of amino groups, which further enhance the adsorption process and encourage the production of hydronium ions, thereby enriching the ionic exchange. The 3D network (Figure 5c) that permits water and ion mobility ensures efficient charge separation due to the created concentration gradients. The interaction of water molecules with the inherent hydroxyl and amino groups within the hydrogel results in the migration of hydronium ions, leading to significant electric potential variation across the material.

The structure and chemical properties of the hydrogel can be controllably regulated by varying the chitosan to vanillin ratio. The output voltages for CM1, CM2, CM3, and CM4 were 0.54, 0.70, 0.71, and 0.72 V, respectively (Figure 5d; Figure S22b, Supporting Information). A higher vanillin content facilitates the generation of more hydrogen bonds and Schiff base linkages (Figure S22c, Supporting Information), leading to the production of a greater amount of H_3_O⁺ upon contact with water, and consequently, a higher output voltage. However, as the vanillin content increased from CM2 to CM4, the excessive amount of vanillin resulted in an increase in the internal resistance of the hydrogel (Figure S22d, Supporting Information). Consequently, CM2 exhibited the highest Isc. Notably, CM3 and CM4 were able to reach maximum output voltage more quickly than CS, CM1, and CM2.Capillary force propels water upward within the hydrogel on the water surface, and this force is determined by pore size and contact angle (Figures S23 and S24, Supporting Information). A decrease in the pore size results in an increase in the capillary force (Figure 5d); this enhanced capillary force accelerates the migration of the water flow through the micropores, enabling the H_3_O^+^ ions to migrate more efficiently along with the water and ultimately resulting in a higher power generation rate.

The performance of the hydrogel is significantly influenced by its thickness and area. Increasing the hydrogel's thickness up to 2 mm, the voltage and current increased due to an increased water gradient and a diffusion potential energy difference within the hydrogel (Figure S25a, Supporting Information). However, once the thickness surpasses 2 mm, ion migration becomes hindered due to the extended diffusion path, resulting in reduced charge separation and diminished the output performance. However, as the area of the CM‐WEG expands, the voltage remains steady while the current increases linearly (Figure S25b, Supporting Information); this is attributed to the increased surface area available for water molecule adsorption and ion exchange.

CM‐WEG also demonstrates exceptional capability for the generation of electrical energy from both simulated seawater and natural seawater (Figure 5f). It produced a steady V_OC_ of 0.77 and 0.84 V, respectively, along with an Isc of 84.2 and 106.1 µA, respectively. Seawater has high conductivity (Figure S26, Supporting Information), implying a higher ion concentration can enhance the flow of electric current and significantly affect the strength of electrical signals.

**Figure 6:**
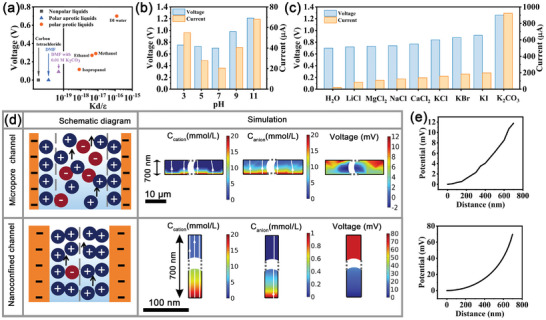
Comprehensive Characterization and Simulation of CM‐WEG Performance. a) Voc of CM‐WEG in polar and non‐polar solutions. Voc and Isc of CM‐WEG in b) solutions with different pH and c) different electrolyte solutions. d) Finite element simulation of ion transport and potential distribution in nanochannel and microchannel. e) Potential variation with distance in the microchannel and nanochannel.

To further understand the WEG mechanism, we used CM‐WEG in various liquid types, as depicted in **Figure** **6**a. In non‐polar and polar protonic solutions, such as carbon tetrachloride and ethyl acetate (DMF), CM‐WEG did not produce any output voltage due to the absence of soluble ions or freely mobile protons. However, addition of K_2_CO_3_ to DMF allowed the CM‐WEG to produce a V_OC_ of 0.09 V, indicating that ions are crucial for voltage generation. Within the polar protic solutions, we observed voltage generation, with the V_OC_ directly proportional to the ratio of the dissociation constant to the dielectric constant. Specifically, the ratio followed the trend: isopropyl alcohol< ethanol< methanol<water. When CM‐WEG was exposed to acidic environments, the abundant hydrogen ions led to increased H_3_O^+^ ions formation, enhancing the electrical output performance (Figure 6b). On the other hand, in alkaline solutions (where the pH was adjusted with NaOH), the migration of Na^+^ ions along with water molecules also improves the electrical output performance (Figure 6b).

The electrical output properties of CM‐WEG exhibited a remarkable enhancement across various electrolyte solutions, highlighting the crucial role of cations. As the dielectric constant of the electrolyte solution declined (Table S2, Supporting Information), its charge‐binding capacity became weaker, enabling a more directional movement of cations and H_3_O^+^ ions together with water molecules; this resulted in a significant increase in both the V_OC_ and Isc of CM‐WEG (Figure 6c). Our experiments have proven that the superior electrical output performance observed on the surface of seawater, compared to pure water, is attributed to the abundance of cations present in the seawater. Notably, in water containing K_2_CO_3_, CM‐WEG achieved an impressive voltage of 1.26 V and a substantial current of 0.922 mA.

The remarkable improvement in electrical output performance is intrinsically associated with the microstructure of the hydrogel. CM hydrogels possess both micropore channels and numerous nanoconfined channels (Figure 6d). To gain a deeper understanding of how these nanochannels and microchannels influence ion transport, a comprehensive analysis was conducted using FEA. When water flows through CM hydrogel, the surfaces of the channels become negatively charged. As the solution passes through the hydrogel's micropore channel, both the cation and anion can be transported, maintaining a cation‐to‐anion ratio that closely resembles the ratio in the liquid, thereby minimizing ion concentration gradients. However, nanoconfined channels with diameters smaller than the Debye length exhibit double electric layer overlap, enabling ion adsorption and screening. Within these nanoconfined channels, anion migration is hindered, whereas cation migration remains unimpeded. Consequently, cation and anion accumulation zones are formed at the entrance and exit of the channels, significantly enhancing the device's performance. As shown in Figure 6e, voltage output increases with K^+^ migration distance, exhibiting a six‐fold increase in nanoconfined channels compared to micropore channels. This demonstrates that nanoconfined channels can selectively transport cations, enhancing electrical output performance. The abundant functional groups and nanoconfined channels of CM enhance migratory abilities to facilitate the migration of hydrated ions and cations contributing to its excellent seawater power generation performance.

### 2.6 Scalability of CM‐WEG and Applications

**Figure 7:**
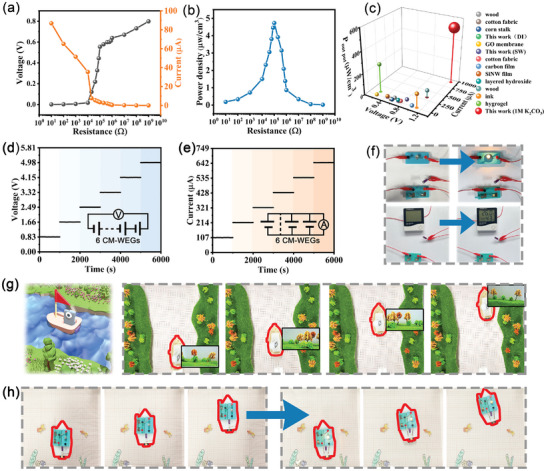
Applications of CM‐WEG. a) The output voltage, current, and b) power density when loading resistance ranges from 10 to10^9^ Ω on seawater surface. c) Hydroelectric power generation benchmark. Schematic of the d) open‐circuit voltage and e) short‐circuit current as a function of integrated CM ‐WEG in series and in parallel, respectively; f) Six CM‐WEG devices can be connected in series to charge a capacitor (0.22 F, 3.3 F); g) CM‐WEG devices can charge capacitors to power a bulb or hygrometer.

The electric output performance of CM‐WEG device on seawater surface was further investigated by connecting various resistors to the device (**Figure** **7**a). As the electric load increased from 10 Ω to 10^9^ Ω, there was an increase in output voltage and a decrease in output current. The maximum output power of 0.946 µW cm^−2^ was achieved at an optimal resistance of 10^5^ Ω (Figure 7b). Notably, CM‐WEG exhibits prolonged and efficient voltage and current output capabilities in deionized water, seawater, and a 1 m K₂CO₃ solution over a span of 10 days (Figure S27, Supporting Information). Compared with previously reported moisture‐ and water‐enabled electricity generation, CM‐WEG demonstrates superior output performance (Figure 7c; Figure S28 and Table S3, Supporting Information). Additionally, the electrical output of the CM‐WEG can be enhanced by integrating multiple devices either in series or in parallel. Voltage output of >4.98 V was obtained by connecting six assemblies in series (Figure 7d), and a current output of ≈647.1 µA was obtained by connecting six assemblies in parallel (Figure 7e). Figure 7f illustrates the effective integration of six devices connected in sequence, resulting in a stable electrical output. CM‐WEG exhibits excellent charging capability, enabling the charging of capacitors to power small bulbs or hygrometers (Figure 7g). The consistent electrical output from CM‐WEG can facilitate efficient energy storage in commercial energy storage systems, thus reducing overall energy consumption.

Monitoring of aquatic environments is crucial for safeguarding human health and fostering ecosystem resilience. CM hydrogels can perform cargo delivery (Figure S29, Supporting Information) and serve as a robust driving module to power a camera‐equipped boat to capture critical hydrological features and document riverside vegetation, which is vital for research (Figure 7h; Movie S7, Supporting Information). Additionally, this hydrogel efficiently converts water into electrical energy, enabling the charging of navigation equipment and thereby facilitating river exploration (Figure 7i; Movie S8, Supporting Information).

## 3 Conclusion

In summary, we have developed a CM hydrogel that exhibits self‐propulsion performance on water surfaces and possesses WEG capabilities. The CM hydrogel robot exhibited rapid movement, long operational life, and considerable velocity. Autonomous navigation on water surfaces was achieved by harnessing the Marangoni propulsion forces generated by a concentration gradient and the dynamic wetting processes facilitated by Schiff base reactions and hydrogen bonding. The locomotion trajectory of the hydrogel can be manipulated by modifying its geometry and adapting to the surrounding environmental conditions. Furthermore, the CM hydrogel displayed exceptional degradability, recyclability, fuel efficiency, and propulsion performance. With its abundant functional groups and nanoconfined channels, the CM‐WEG facilitates efficient water absorption, rapid ion transport, and selective ion transport, ultimately enhancing electricity generation; it spontaneously generates high voltages of 0.83 and 1.26 V on the surfaces of seawater and water containing K_2_CO_3_, respectively. CM‐WEG can potentially generate substantial electricity, particularly in electrolyte solutions with low dielectric constants and high cation concentrations. This innovative hydrogel can function as a mobility module, enabling programmed movement and diverse applications such as cargo delivery, while also enabling equipment charging. This study establishes the foundation for developing custom‐designed hydrogels and provides a strategic framework for constructing self‐powered and self‐propelled soft robots.

## Conflict of Interest

The authors declare no conflict of interest.

## Supporting information



Supporting Information

Supplemental Video 1

Supplemental Video 2

Supplemental Video 3

Supplemental Video 4

Supplemental Video 5

Supplemental Video 6

Supplemental Movie 7

Supplemental Movie 8

## Data Availability

The data that support the findings of this study are available from the corresponding author upon reasonable request.
