# Supplementary material for: High‐Performance MXene Hydrogel for Self‐Propelled Marangoni Swimmers and Water‐Enabled Electricity Generator
Source: Adv Sci (Weinh). 2024 Nov 18;12(2):2408161. doi: 10.1002/advs.202408161 (PMC11727138; doi:10.1002/advs.202408161)
Supplement: Supplementary file 1 — Supporting Information [file ADVS-12-2408161-s004.docx]

**Supporting Information**

**High-Performance MXene Hydrogel for Self-Propelled Marangoni Swimmers and Water-Enabled Electricity Generator**

Jiayi Zhou^a^, Yan Zhang^a^*, Ming Zhang^a^, Dongye Yang^a*^, Wenwei Huang^a^, Ao Zheng^b^, Lingyan Cao^b*^

^a^ School of Material Science and Engineering, Shanghai University of Engineering Science, Shanghai 201620, P.R. China;

^b^ Department of Prosthodontics, Shanghai Ninth People's Hospital, Shanghai Jiao Tong University School of Medicine; College of Stomatology, Shanghai Jiao Tong University, Shanghai 200011, P.R. China.

Corresponding author: yanzhang@sues.edu.cn (Yan Zhang),

dongye.yang@sues.edu.cn (Dongye Yang),

cly_linya@163.com (Lingyan Cao)

**List of contents**

**Supplementary Notes…………………………………...……………………………3**

**Supplementary Figures………………………………………………………………7**

**Supplementary Tables………………………………………………………………23**

**Supplementary References…………………………………………………………25**

**Supplementary Notes**

**Experimental section**

*Chemicals and Materials:* Ti_3_AlC_2_ powder was obtained from 11 Technology Co. Ltd (Jilin, China). Lithium fluoride (LiF), hydrochloric acid (37%), ethanol (AR, 99.7%), glacial acetic acid (99%), chitosan (Degree of deacetylation ≥ 95%, viscosity is 100-200 mPa•s) and vanillin (99%) were purchased from Aladdin (Shanghai, China). A solution of graphene oxide (GO) was prepared from natural graphite powder using an improved Hummers method. The Ti_3_C_2_Tx MXene dispersion was synthesized based on previous reports.

*Preparation of Hydrogels:* 50 mg of MXene was mixed with a chitosan solution (made from 0.4 g of chitosan and 6 ml of 1% acetic acid) and then sonicated for 15 minutes. Next, 2 ml of a 0.12 g/ml vanillin solution was added to the mixture. The mixture was then poured into a poly (methyl methacrylate) mold and left for one day, resulting in a hydrogel named CM2. For comparison, CM hydrogels with various ratios of chitosan and vanillin (10:5, 10:7, 10:8) were prepared and named as CM1, CM3, and CM4, respectively. Unless otherwise specified in the work, CM refers to CM2. A CS hydrogel was prepared the same way without MXene, and a CG hydrogel was prepared using GO instead of MXene. FTCMs were created by freezing the MXene-chitosan-vanillin mixture in a PMMA mold at -20 °C for 20 hours, followed by thawing at room temperature for 4 hours. After three cycles, the FTCM was produced.

*Repeated Loading:* After self-propelling was complete, the hydrogel was submerged in a 5% (w/v) vanillin-ethanol solution for one hour. The treated hydrogel was subsequently washed with deionized water to remove any unreacted vanillin residues.

*FEA simulation:* The numerical analysis of the vanillin distribution surrounding the hydrogels was performed using the transient diffusion module in COMSOL Multiphysics 6.0. The simulation computed the velocity and trajectories of the hydrogels by applying simulated forces through the multibody dynamic module. Additionally, the electrostatic module and the dilute substance transfer module were used to simulate and calculate the distribution of anions, cations, and voltage within the microchannel and nanoconfined channel.

*Fabrication of CM-WEG:* The CM (20 mm × 10 mm × 2 mm) was utilized as a source of electricity generation, with commercially available copper electrodes serving as the bottom and upper electrodes of the device, which was encapsulated with commercial biaxially oriented polypropylene (BOPP) tape. To measure the electrical performance, an instrument from Keithley Instruments (Model 6514) was used for both open-circuit voltage and short-circuit current tests.

**Calculate of capillary force theoretically and surface tension**

Based on Kralchevsky and Nagayama ^[1]^ solving the Laplace equation of capillarity under the assumption that the liquid surface deformation is not very large, the transverse capillarity force is theoretically calculated using the Eq. (1):

$F\approx{-2\pi{\gamma Q}_{1}Q_{2}}/L$ (S1)

where Q_i_=r_i_sinψ_i_ (i=1, 2) are the so-called “capillary charges”, and r_i_ and ψ_i_ are the radius of the contact line and the slope angle at the contact line of the respective particle.γ is the surface tension of the solution, L is the distance between motor and the boundary.

According to the Marangoni propulsion theory ^[2]^, the theoretical driving force obtained from the Marangoni effect induced by the release of vanillin to produce a surface tension gradient is calculated.

Typically, force Fs can be obtained by

$F_{S}=\gamma_{2}L_{2}-\gamma_{1}L_{1}$ (S2)

Where γ_1_ is the surface tension of the fluid 5 mm near the hydrogel, γ_2_ is the surface tension of the fluid at the vanillin diffusion boundary; L_1_ is the fixed distance 5 mm, and L_2_ is the radius of the vanillin diffusion boundary.

**Calculate of** **thermal Marangoni surface tension**

The temperature gradient during the motion were further analyzed using the AnalyzIR. The thermal Marangoni surface tension gradient induced by the temperature was calculated using the formula ^[3]^:

γ=b_0_+b_1_T+b_2_T^2^ (S3)

Where γ is the local surface tension; b (b0 = 75.796 mN/m, b1 = -0.145 mN/(m*°C), b2 = -0.00024 mN/(m*°C^2^) are constants; T is temperature (°C).

**Calculate of capillary pressure**

The capillary pressure was calculated using the formula:

$P_{C}=\frac{2\gamma cos\theta}{r}$ (S4)

Where Pc is the capillary pressure, γ is the local surface tension, $\theta$ is the contact angle, r is the radius.

**Calculate of ionic conductivity**

Ionic conductivity was calculated according to the formulas:

$\sigma=\frac{L}{R*S}$ (S6)

Where $\sigma$ is the ionic conductivity; R is the resistance of electrodes; S is the cross-sectional area; L is the length.

**Calculate of the relative content of Schiff-based bonds**

The relative content of Schiff-based bonds in the hydrogels was calculated using the integration method ^[4]^.

**Supplementary Figures**

**
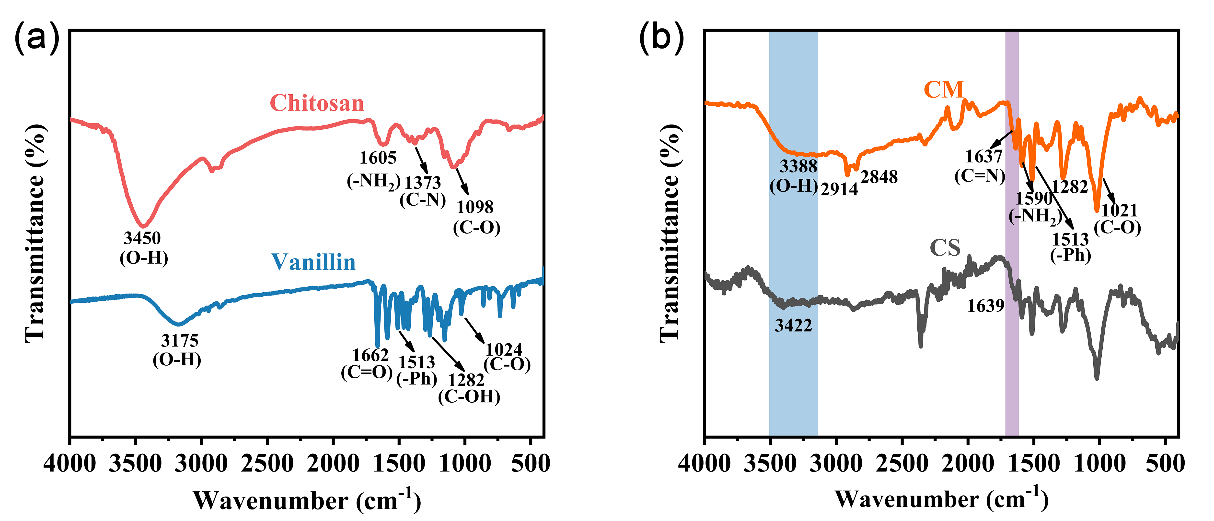
**

**Figure S1.** (a) FTIR spectra of chitosan and vanillin, respectively. (b) FTIR spectra of CS and CM hydrogel, respectively.

The chitosan (red line) shows the characteristic peaks at 3450, 1605, 1373 and 1098 cm^−1^ corresponding to the stretching vibration of hydroxyl groups, the symmetric bending vibration of primary amine groups, the stretching vibration absorption of C-N bonds and the stretching vibration absorption peak of C-O bonds in -CH₂-OH, respectively ^[5]^. The vanillin (blueline) shows the characteristic peaks at 3175, 1662, 1513, 1282 and 1024 cm^−1^ corresponding to the stretching vibration of hydroxyl groups, the stretching vibration of aldehyde groups, the benzene ring groups, the bending vibration of phenolic hydroxyl groups and the stretching vibration of C-O bonds in methoxy groups, respectively ^[6]^.





**Figure S2.** The spinning velocity of the CM hydrogel robots.


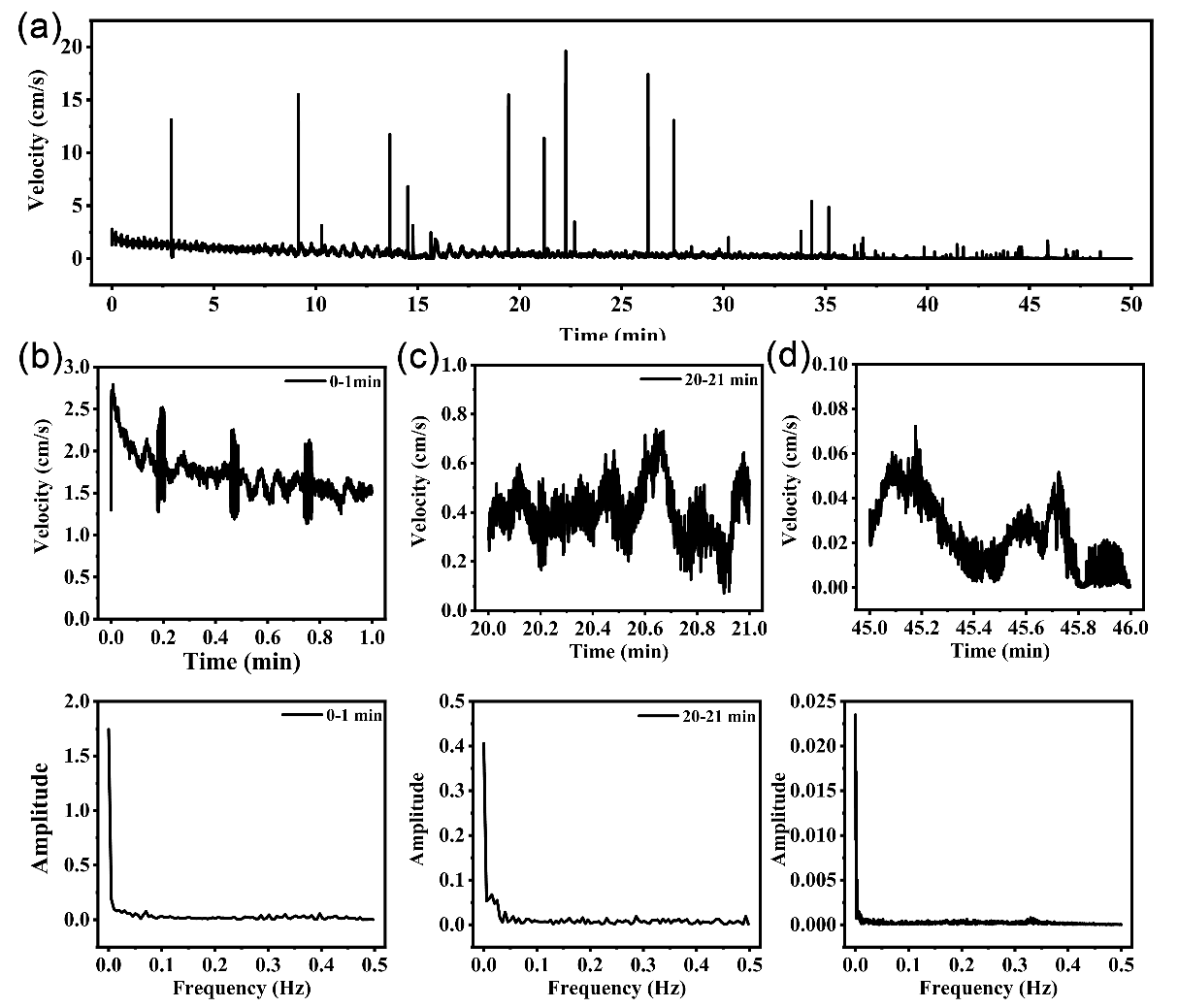


**Figure S3.** Instantaneous velocity of hydrogel and the frequency of velocity changes. (**a**) Instantaneous velocity of the CM hydrogel robot during the whole locomotion. (**b**) Instantaneous velocity of hydrogel and the frequency of velocity changes from 0 to 1 minute. (**c**) Instantaneous velocity of hydrogel and the frequency of velocity changes from 20 to 21 minute. (**d**) Instantaneous velocity of hydrogel and the frequency of velocity changes from 45 to 46 minute.

**Figure S4.** FTIR spectra of SCM hydrogel.

The relative content of Schiff-based bonds in CM and SCM hydrogel is 61.44% and 52.56%, which demonstrates that rupture of dynamic bonds.


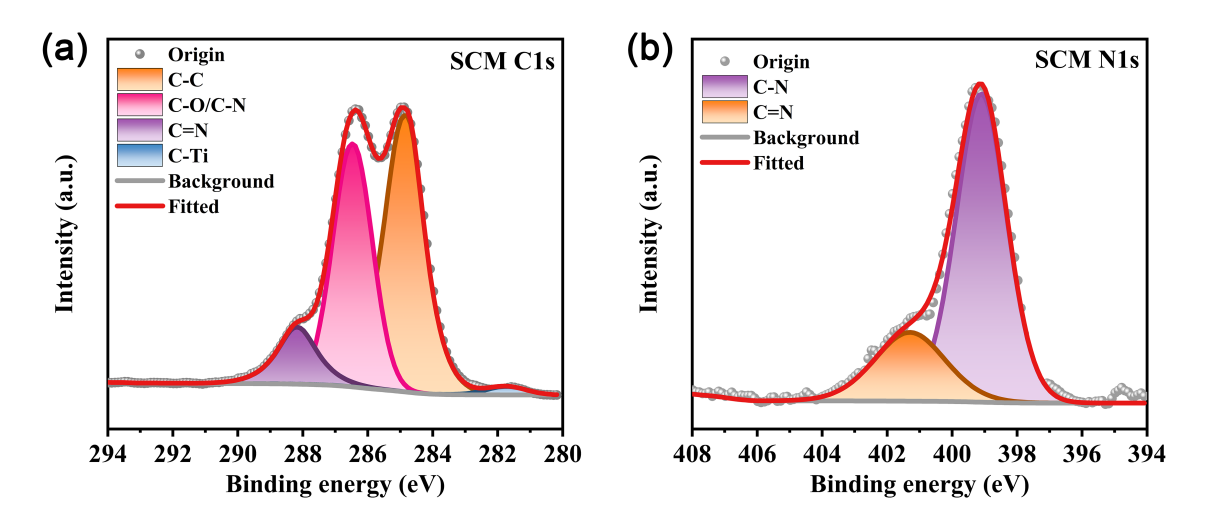


**Figure S5.** (a) C1s XPS spectra and (b) N1s XPS spectra of swollen CM (SCM) hydrogel, respectively.


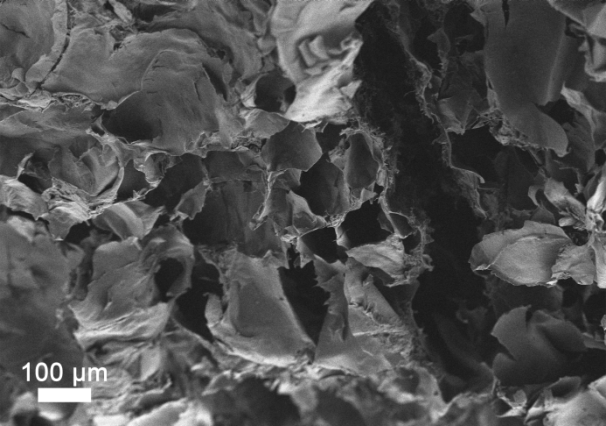


**Figure S6.** SEM image of the SCM hydrogel.


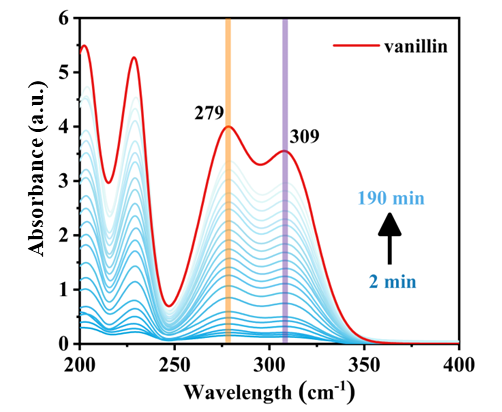


**Figure S7.** The UV spectra of the solution after hydrogel locomotion.


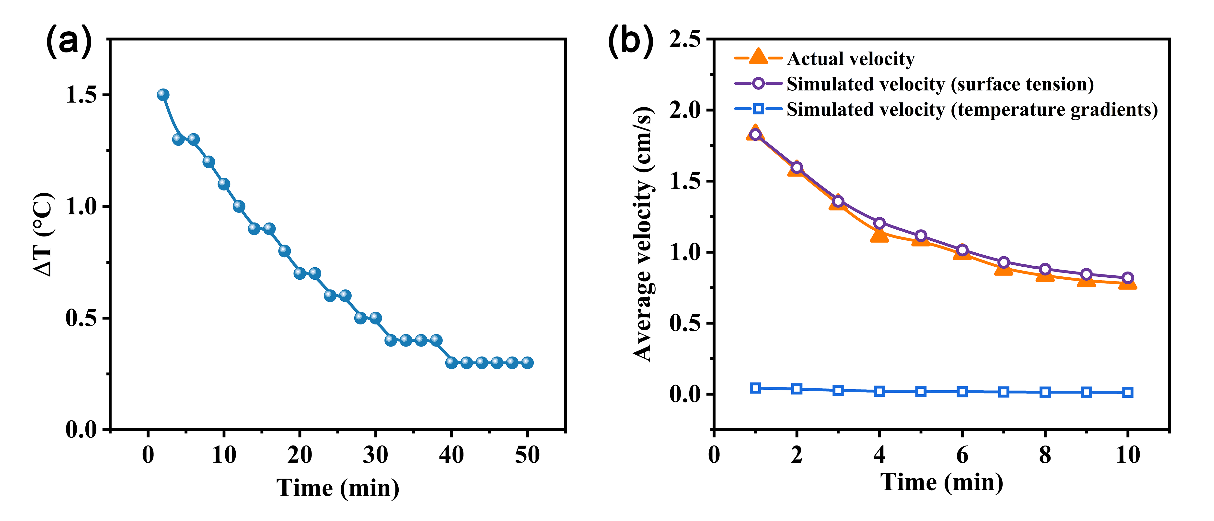


**Figure S8.** Surface tension of CM hydrogel. (a) The temperature gradient during locomotion. (b) The average velocity of actual velocity and simulated velocity.

The temperature gradient during the motion were further analyzed using the AnalyzIR. After two minutes, the temperature gradient resulting from the release of 0.22 mg of vanillin was measured at 1.5 °C. The surface tension gradient induced by the temperature was 0.016 mN/m, which indicates that temperature gradient generated during the motion of the hydrogel has a minimal impact on its overall movement.

The velocity simulated based on the surface tension induced by concentration was consistent with the trends observed in the actual velocity. In contrast, the velocity simulated based on the surface tension induced by temperature was significantly lower and can be disregarded.


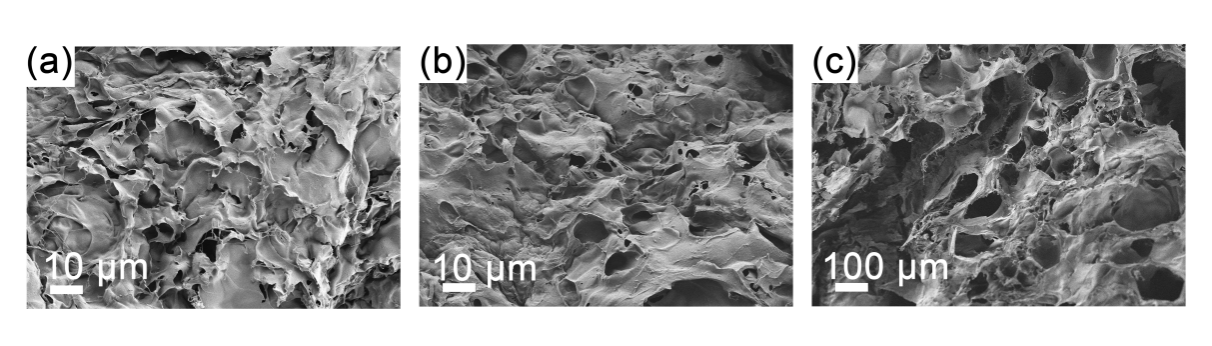


**Figure S9.** SEM image of CS, CG and FTCM hydrogel, respectively.

**Figure S10.** FTIR spectra of CG and FTCM hydrogel, respectively.


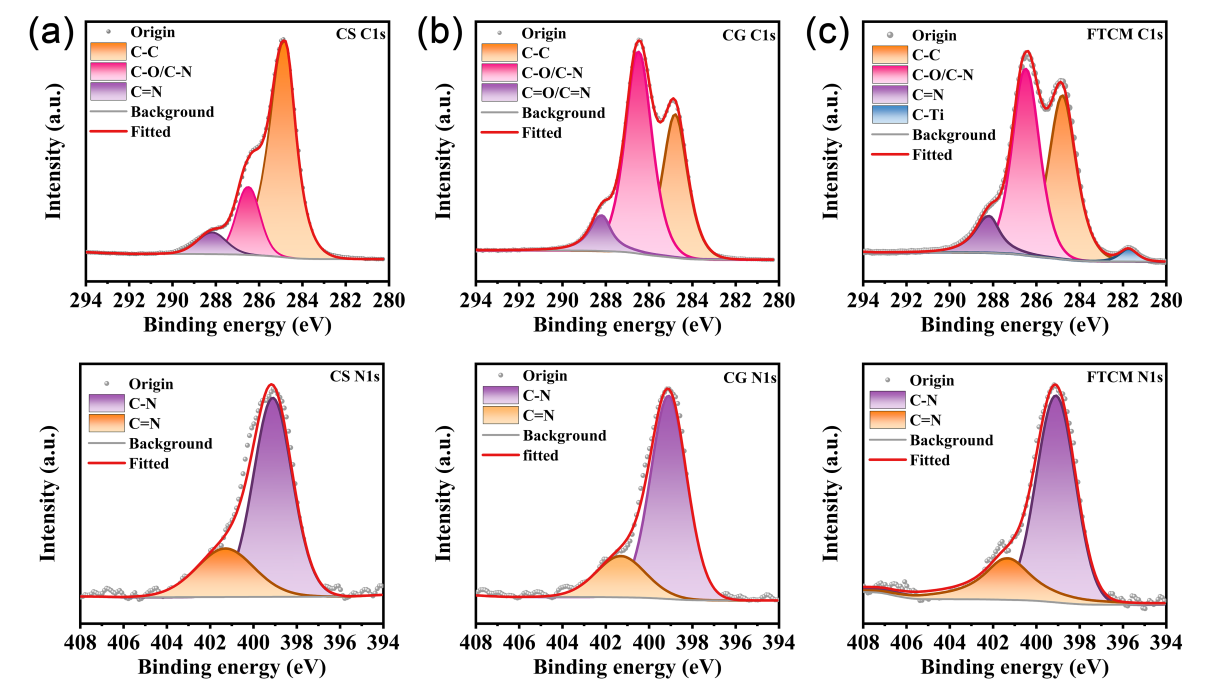


**Figure S11.** XPS spectra of the hydrogels. (**a-c**) C 1s XPS spectra of the CS, CG and FTCM hydrogel, respectively. (**d-f**) N 1s XPS spectra of the CS, CG and FTCM hydrogel, respectively.

**Figure S12.** The vanillin release of the CS, CG, CM and FTCM hydrogel, respectively.


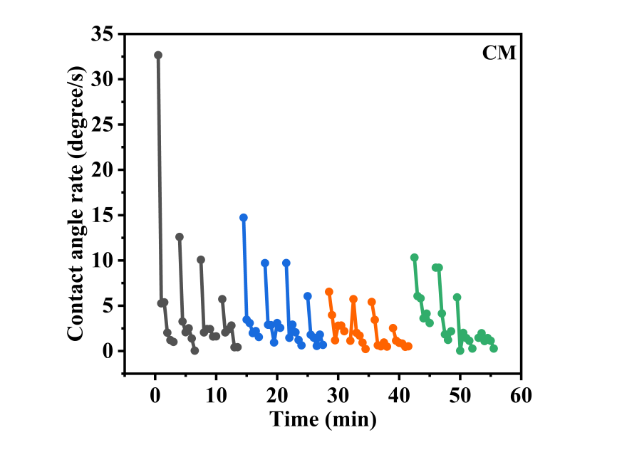


**Figure S13.** The contact angle change rate of CM hydrogel.


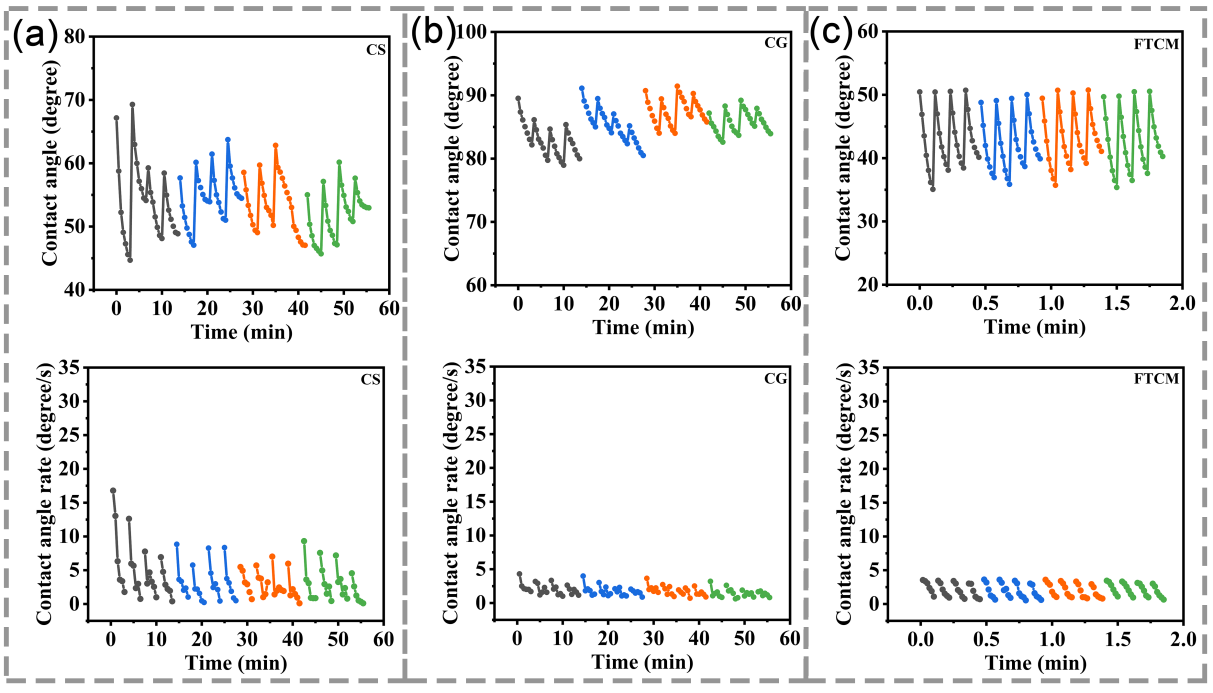


**Figure S14.** The contact angle changes and the contact angle change rate of CS, CG and FTCM hydrogel, respectively.


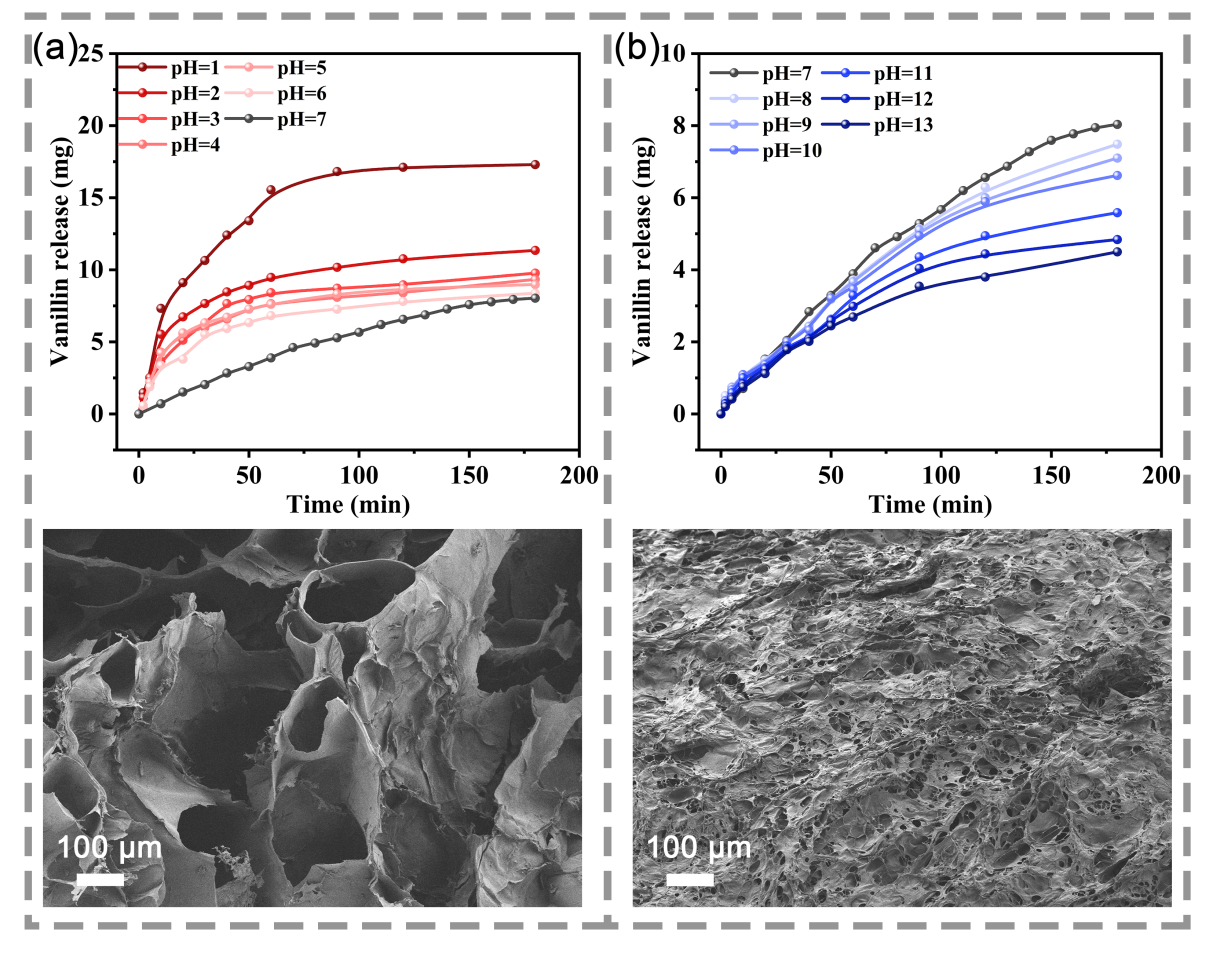


**Figure S15.** The vanillin release and the SEM image of CM hydrogel robots on aqueous solutions with different pH values. (**a**) The vanillin release and the SEM image of the CM hydrogels under acidic conditions. (**b**) The vanillin release and the SEM image of the CM hydrogels under alkaline conditions.


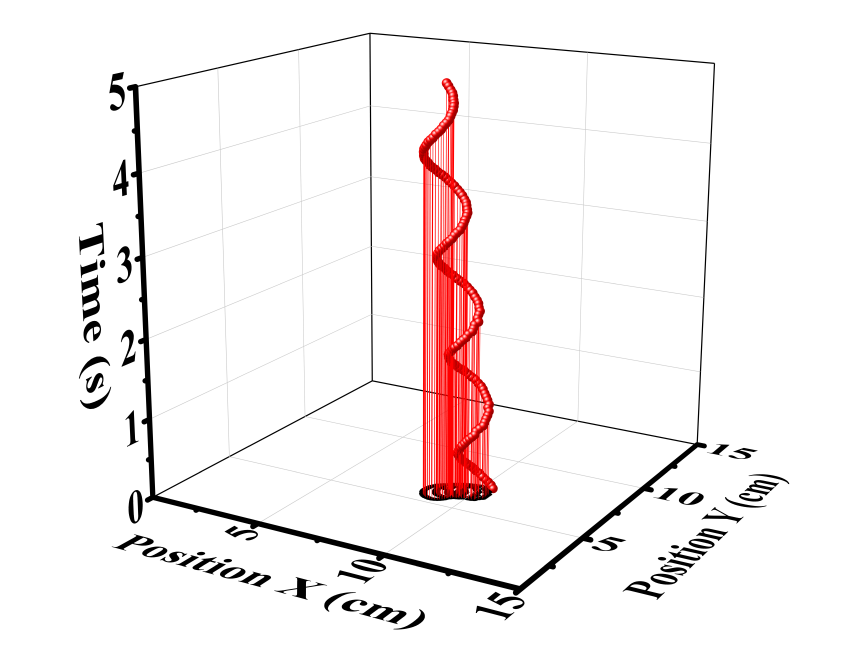


**Figure S16.** The locomotion trajectory of a square CM robot.





**Figure S17.** The initial velocity and the motion time of CM hydrogel robots with different L/w ratios.


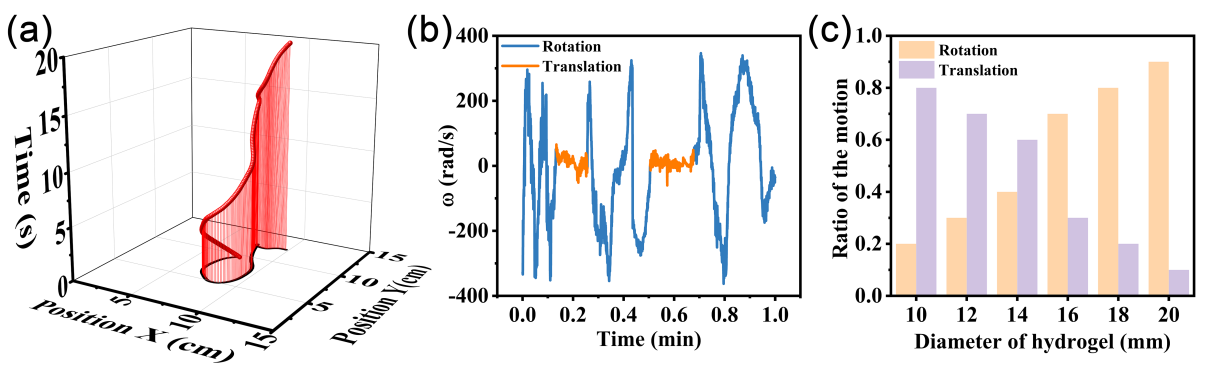


**Figure S18.** Locomotion of circular CM robots. (**a**) The locomotion trajectory of a circle CM robot (**b**) The spinning velocity of the circle CM robot. (**c**) Ratio of circular CM robots taking rotation motion or rotation motion changing with diameters of hydrogels.


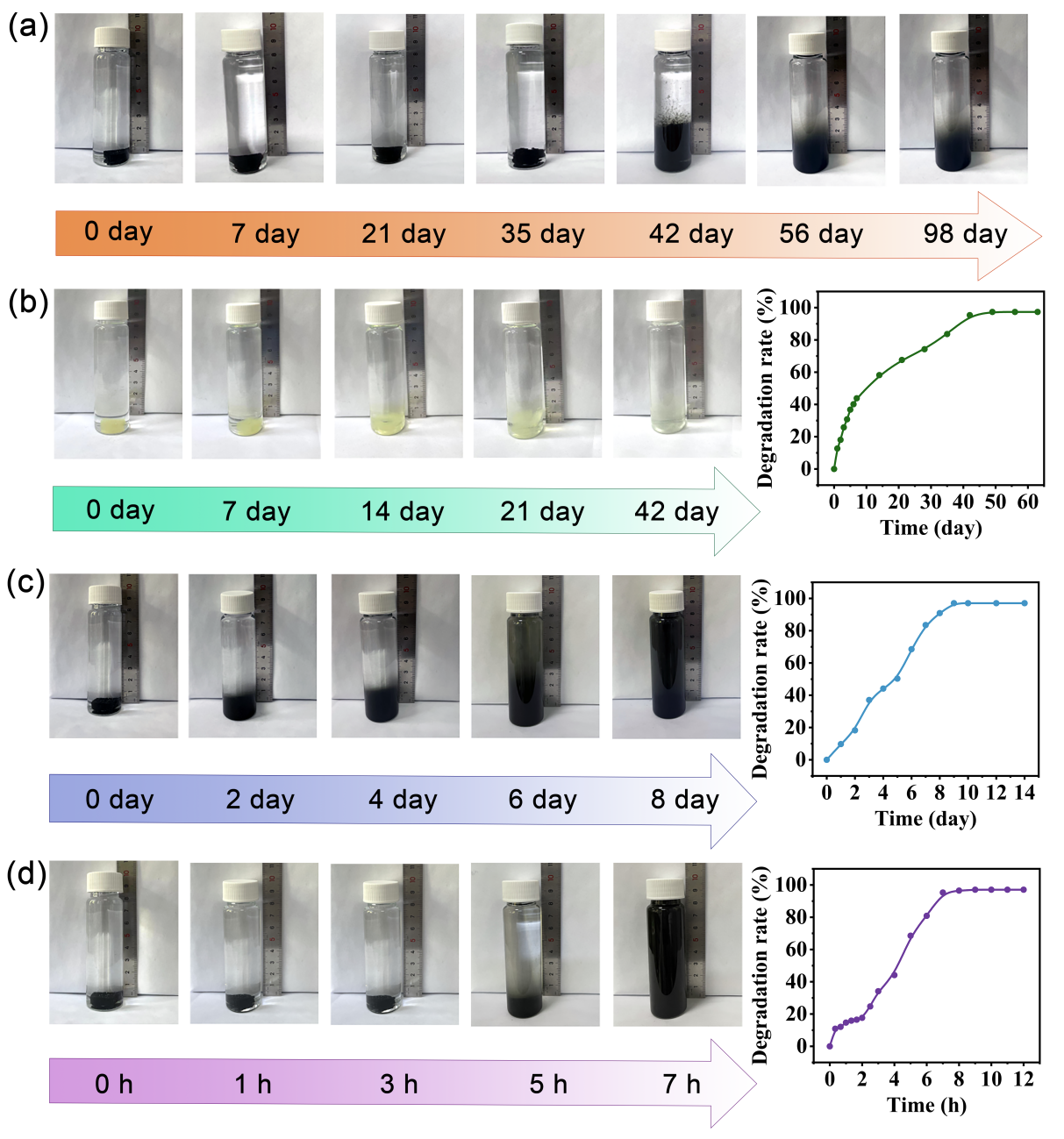


**Figure S19.** The degradation of the hydrogels. (**a**) Photographs of the CM hydrogel placed in DI water for different times. (**b**) Photographs and the degradation rate of the CS hydrogel placed in deionized water for different times. (**c**) Photographs and the degradation rate of the CM hydrogel placed in aqueous solution with pH=6 for different times. (**d**) Photographs and the degradation rate of the CM hydrogel placed in aqueous solution with pH=1 for different times.


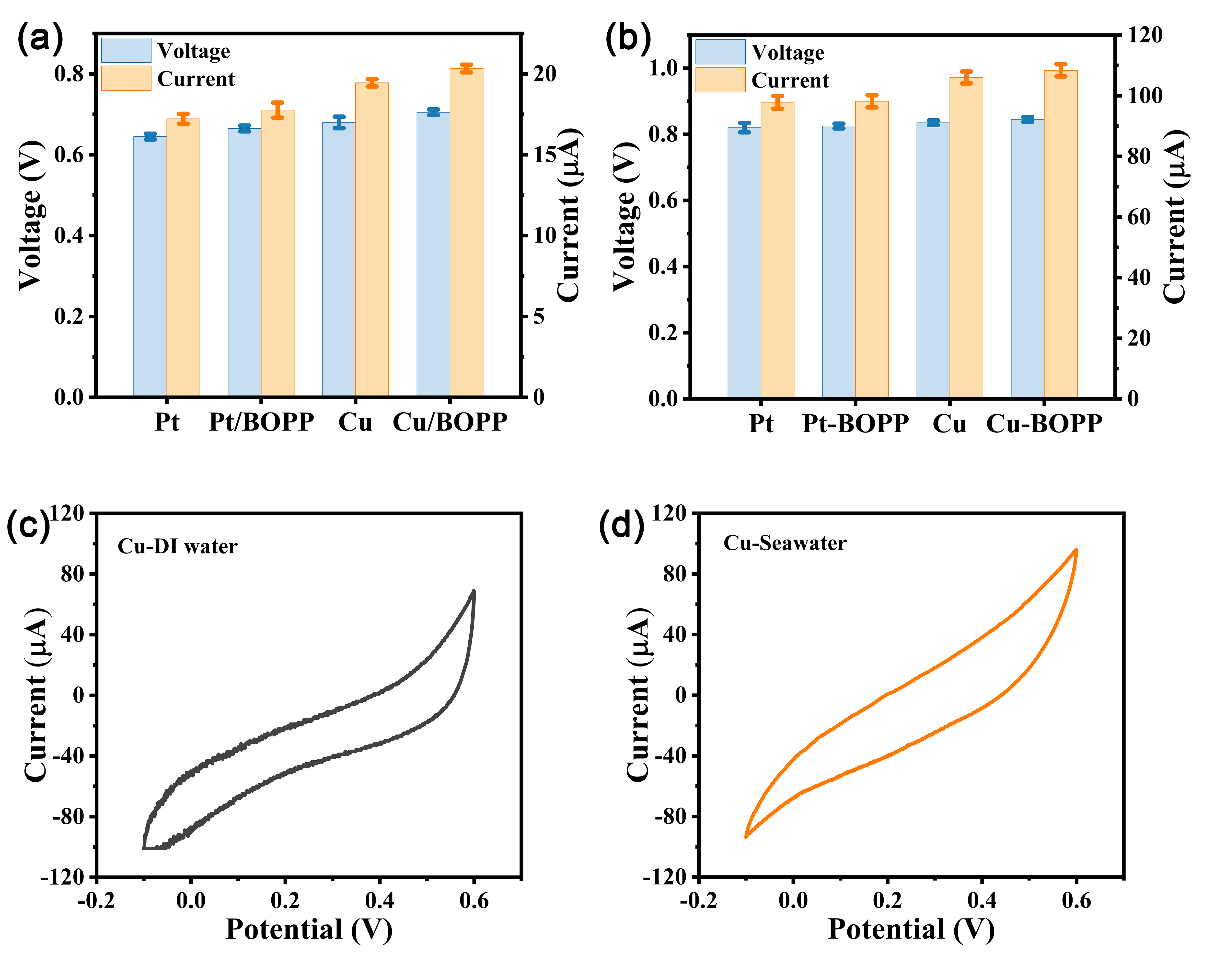


**Figure S20.** The performance of CM-WEG. Voltage and current output of CM-WEG with different electrodes obtained by placing the CM-WEG on (a) DI water and (b) seawater. CV curves of CM-WEG with Cu electrodes obtained by placing the devices on (c) DI water and (d) seawater.

To prevent corrosion of the metal electrodes by seawater, the electrodes were encapsulated with BOPP tape, and there was no significant difference in the output performance of the device. The output voltage and short-circuit current using the platinum electrode when placing on DI water or seawater were slightly lower than those obtained with copper electrodes. Figures S20(c-d) show the CV curves of the device measured using the copper electrode in both deionized water and seawater. No redox-related peaks were found, excluding the possibility of redox reactions occurring at the metal electrodes ^[7]^.


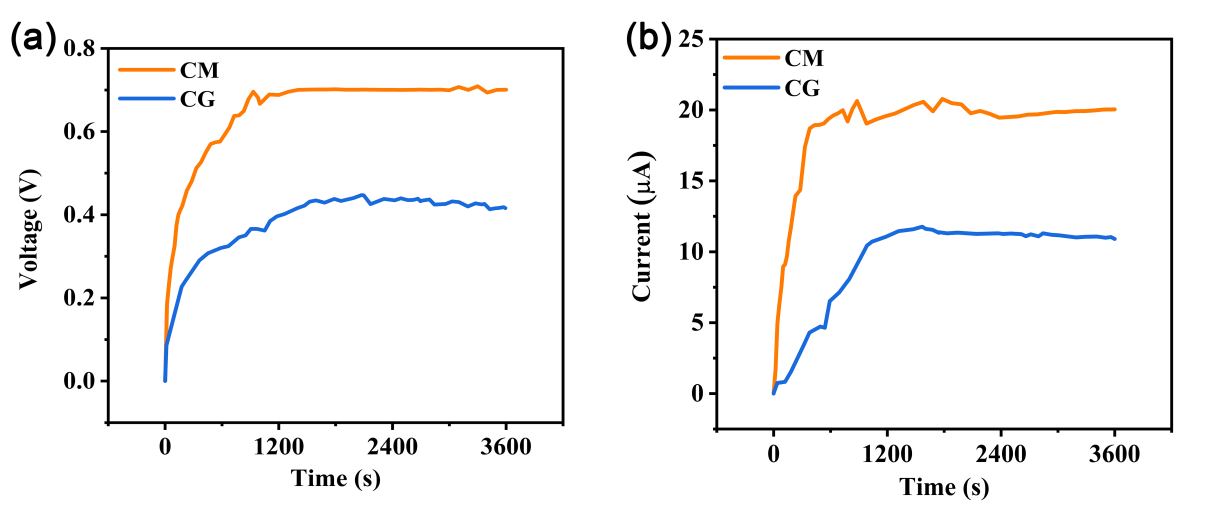


**Figure S21.** The performance of CM-WEG and CG-WEG. (a) The output voltages of CM and CG. (b) The short-circuit currents of CM and CG hydrogel.


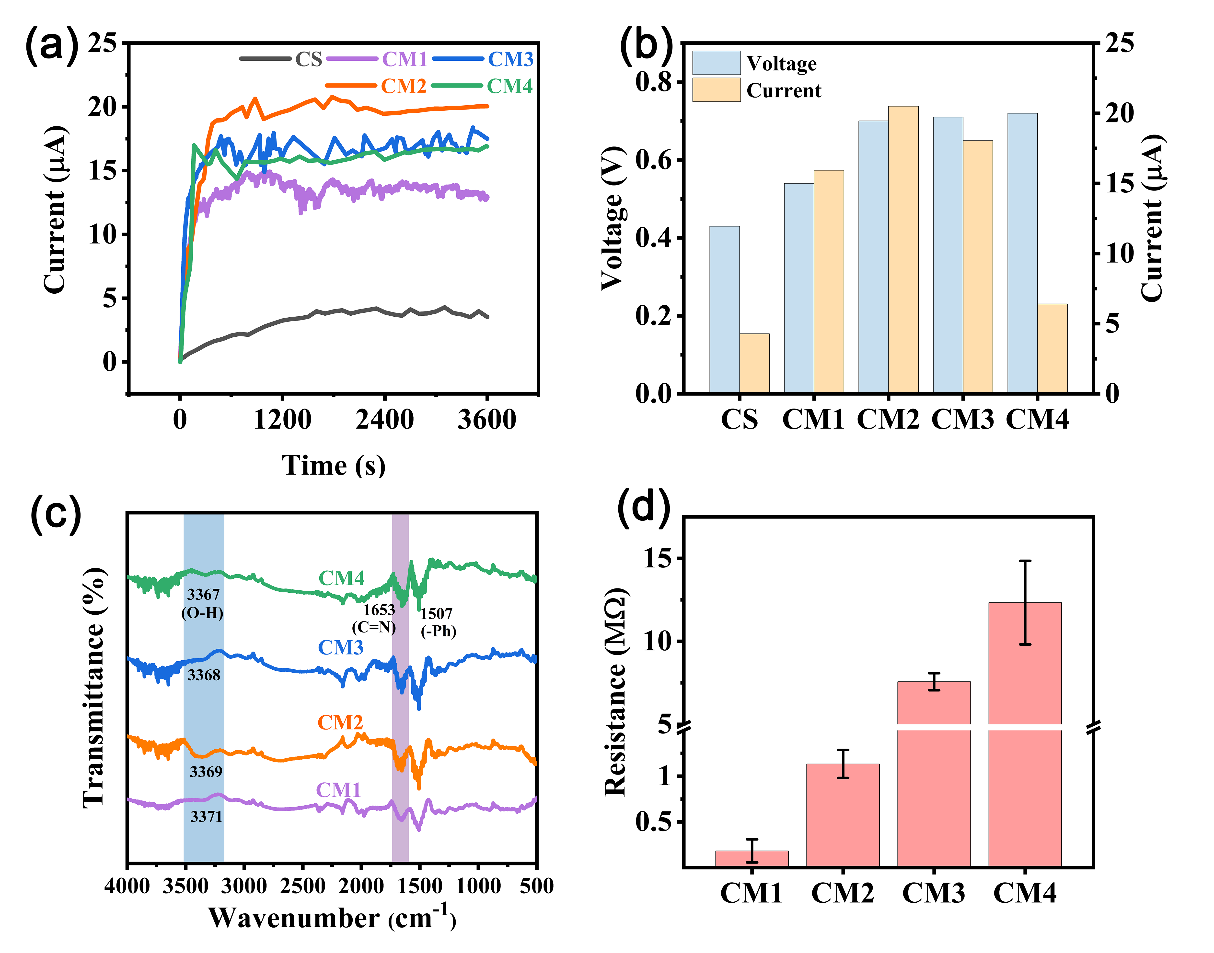


**Figure S22.** Performance of CS and CM1-CM4 hydrogels. (a) The current, (b) the peak output voltage and current of CS and CM1-CM4 hydrogels, respectively. (c) The FTIR spectra and (d) internal resistance of CM1-CM4 hydrogels, respectively.


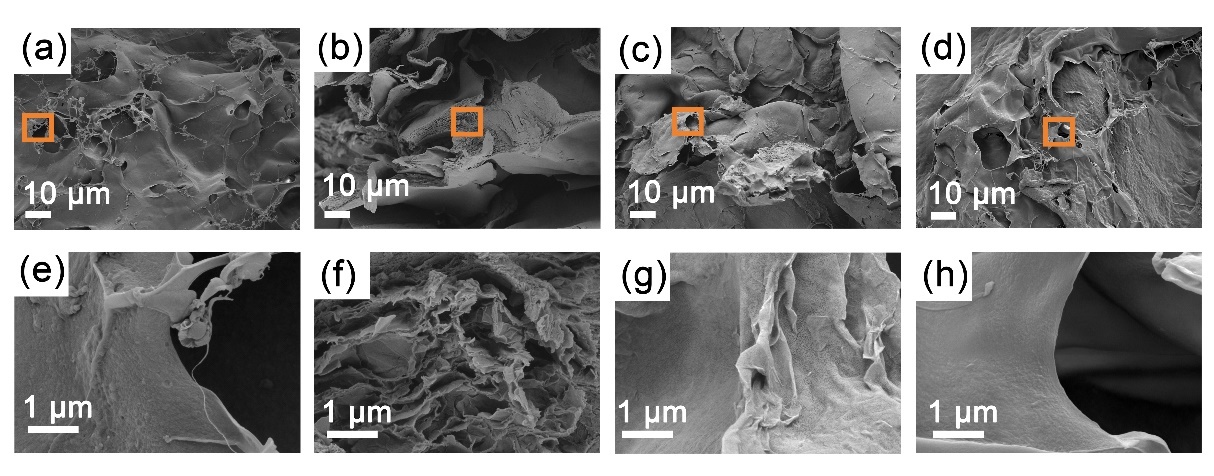


**Figure S23.** (a-b) SEM images of CS, CM1, CM3 and CM4 hydrogels (scale bars are 10 μm). (e-f) SEM images of CS, CM1, CM3 and CM4 hydrogels (scale bars are 1 μm)


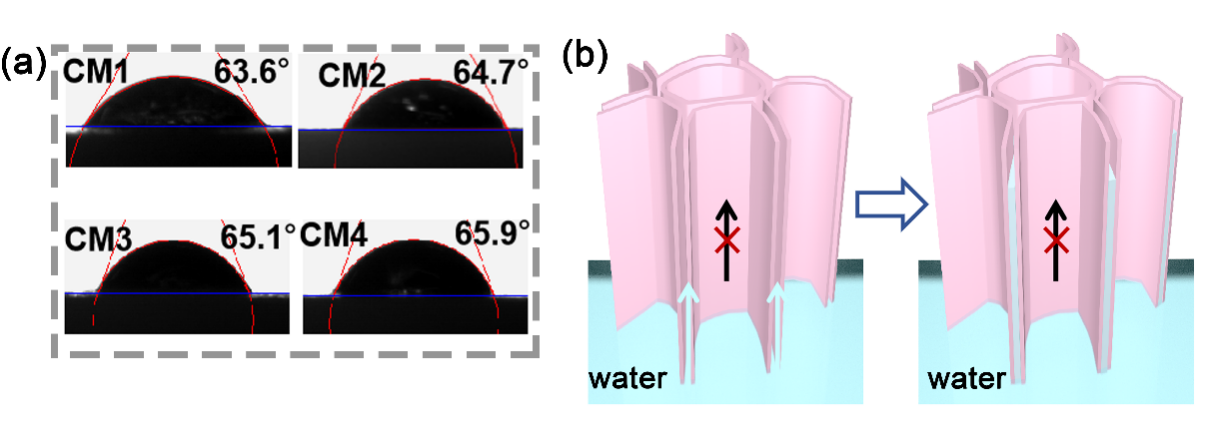


**Figure S24.** The contact angle of CM1-CM4 hydrogels, respectively.


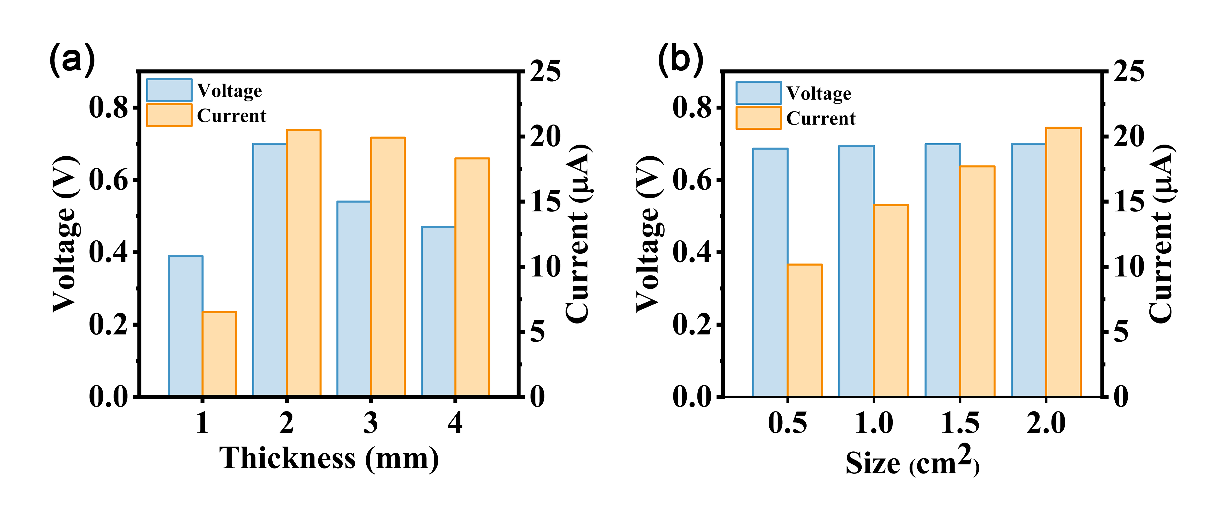


**Figure S25.** Voltage and current output of CM-WEG with different (a) thickness and (b) size.


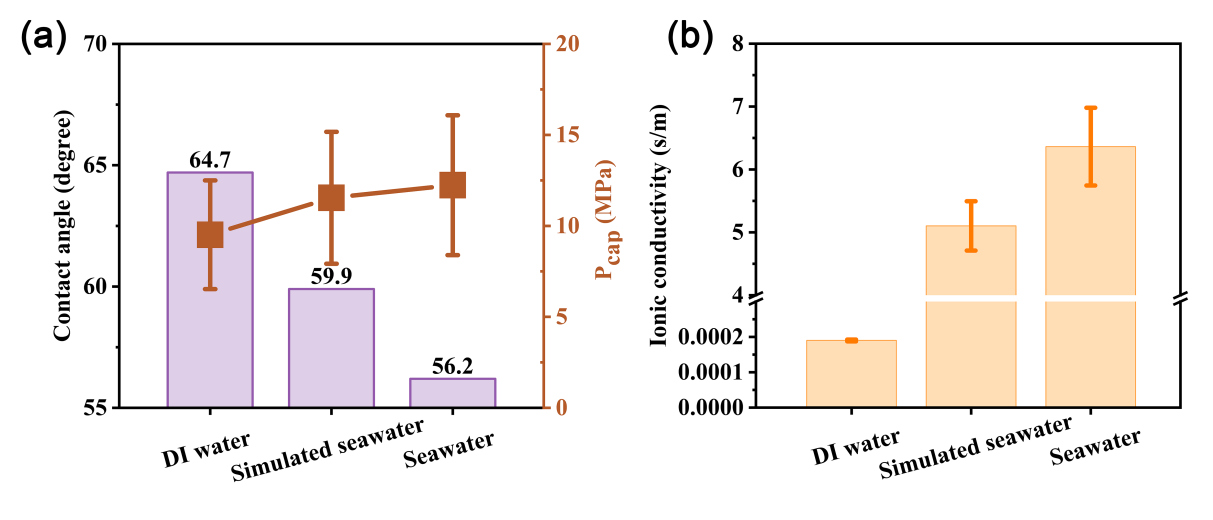


**Figure S26.** Comparison of DI water, simulated seawater and actual seawater. (a) The contact angle, and theoretical capillary pressure of CM hydrogel. (b) The ionic conductivity of DI water, simulated seawater and actual seawater.

**
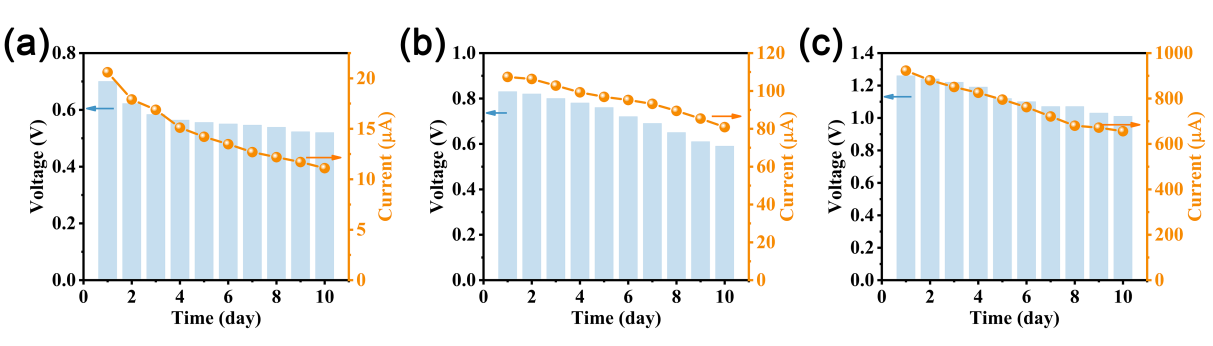
**

**Figure S27.** Long-term voltage and current testing of CM-WEG on deionized water (a), natural seawater (b), and 1 M K_2_CO_3_ solution (c), respectively.


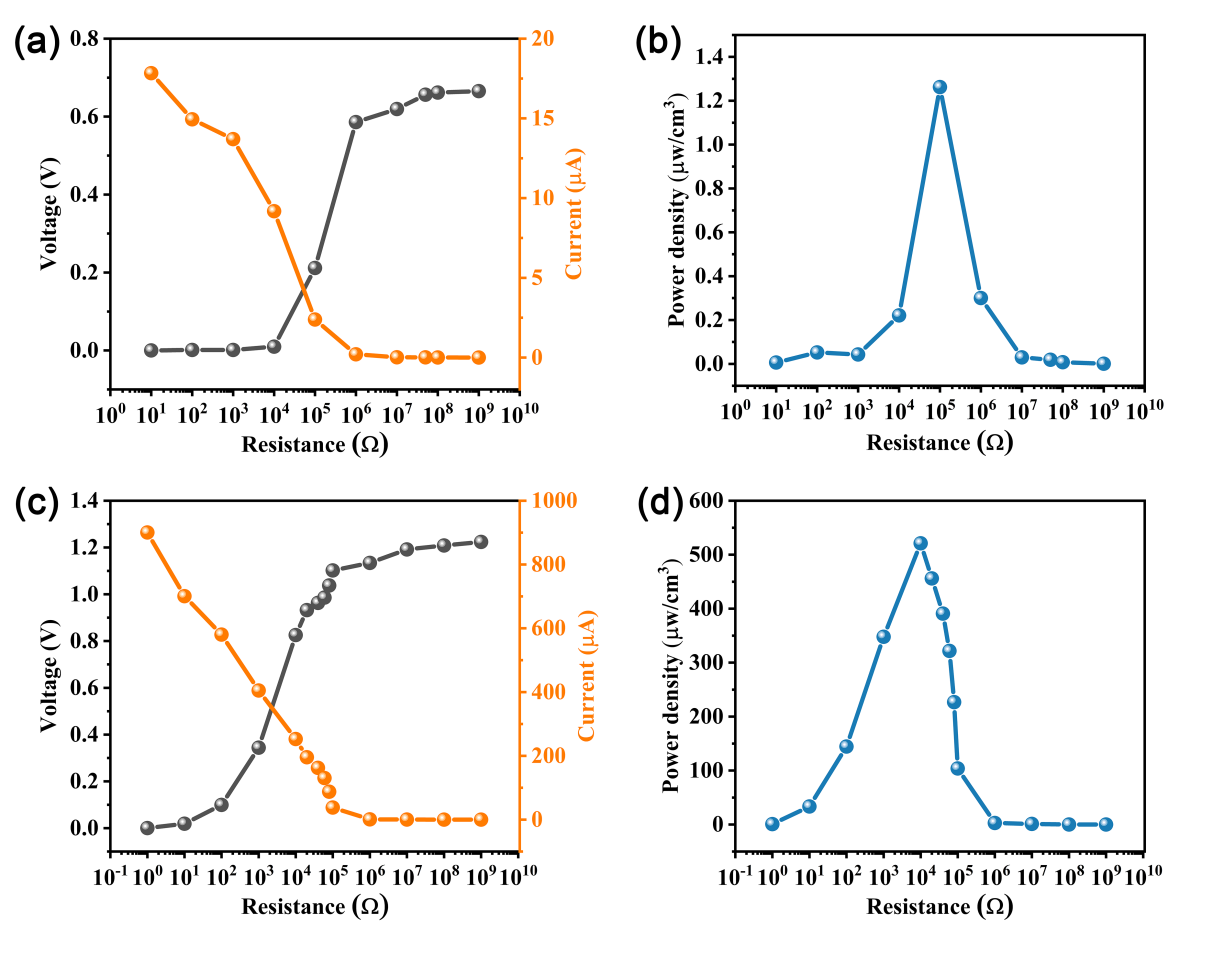


**Figure S28.** (a) The output voltage, current and (b) power density when loading resistance ranges from 10 Ω to10^9^ Ω in DI water. (c) The output voltage, current and (d) power density when loading resistance ranges from 1 Ω to10^9^ Ω in 1 M K_2_CO_3_ solution.


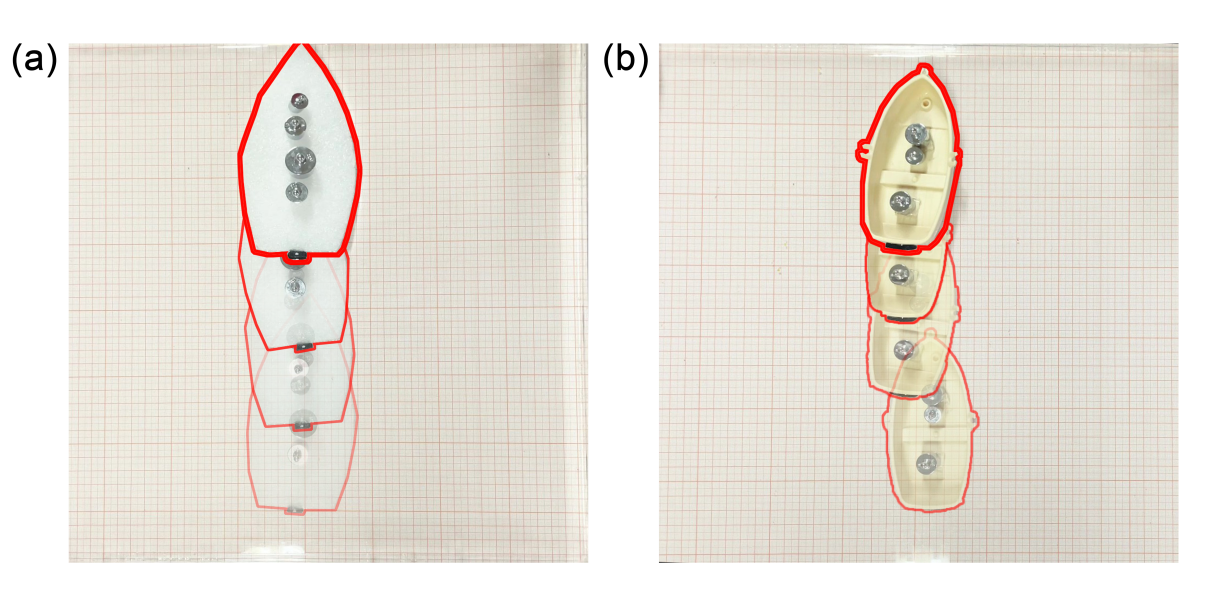


**Figure S29.** Cargo delivery. A 0.32 g hydrogel drives (a) a 1 g foam boat carrying a load of 100 g or (b) a 32 g plastic boat with a load of 50 g.

**Supplementary Tables**

**Table S1. Self-Propelled robot benchmarking.**

| Motor type | Fuel economy (min/g) | Velocity output (mm s^-1^)/mm^3^ | degradability | Recyclability | Repeatability | Reference in text |
| --- | --- | --- | --- | --- | --- | --- |
| Camphor boats | 4.23 | 7.96 | / | / | / | [8] |
| Camphor boats | 58.47 | 39.62 | / | / | / | [9] |
| Camphor boats | 58.34 | 1.11 | / | / | / | [10] |
| Camphor boats | 77.78 | 0.49 | / | / | / | [11] |
| droplets/particles | 2167.67 | 250 | / | / | / | [12] |
| droplets/particles | 1000 | 20.00 | / | / | / | [13] |
| droplets/particles | 3917 | 280 | / | / | / | [14] |
| droplets/particle | 272.27 | 200 | / | / | / | [15] |
| porous materials, MOF | 565.21 | 30 | / | / | / | [16] |
| porous materials, MOF | 1300 | 200 | / | / | / | [17] |
| porous materials, fiber | 117 | 75 | / | 5 | / | [18] |
| porous material, capsule | 2660 | 203.74 | / | / | / | [19] |
| hydrogels | 800 | 20 | / | / | / | [20] |
| hydrogels | 1500 | 30 | / | / | / | [21] |
| hydrogels | 3000 | 260 | / | / | / | [22] |
| hydrogels | 4167 | 63.6 | / | / | / | [23] |
| hydrogels | 400 | 4.88 | / | / | / | [24] |
| CM hydrogel robot | 5303.37 | 310.4153 | √ | 25 | √ | This work |

**Table S2.** The dielectric constant of different substances.

| electrolyte | H_2_O | LiCl | MgCl_2_ | NaCl | CaCl_2_ | KCl | KBr | KI | K_2_CO_3_ |
| --- | --- | --- | --- | --- | --- | --- | --- | --- | --- |
| dielectric constant | 80 | 11.1 | 9.7 | 6.1 | 6.6 | 5.9 | 5.6 | 4.8 | 4.6 |

**Table S3.** Hydroelectric power generation benchmark

| Material | Hydroelectric power generation | Voltage (V) | Current (μA) | P_out put_ (μW/cm^3^) | Discharge durable time (h) | Hourly voltage decay rate (%) | Hourly current decay rate (%) | Reference in text |
| --- | --- | --- | --- | --- | --- | --- | --- | --- |
| wood | moisture | 0.57 | 62.6 | 0.71 | 26 | 0.81 | 3.68 | [25] |
| cotton fabric | water | 0.53 | 3.91 | 0.80 | 1.39 | 65.21 | 72 | [26] |
| corn stalk | moisture | 0.56 | 3.6 | 0.88 | 192 | 0.06 | / | [27] |
| This work（DI water） | **water** | **0.7** | **20.5** | **1.26** | **240** | **0.11** | **0.19** | **This work** |
| GO membrane | moisture | 0.205 | 0.036 | 1.53 | 100 | 0.83 | / | [28] |
| This work （seawater) | **water** | **0.83** | **107.4** | **4.75** | **240** | **0.12** | **0.10** | **This work** |
| cotton fabric | moisture | 0.74 | 22.5 | 6.17 | 8.33 | 0.97 | 7.73 | [29] |
| carbon film | water | 1 | 0.6 | 8.1 | 480 | 0.13 | 0.23 | [30] |
| SiNW film | water | 0.45 | 10.2 | 14.2 | 1.39 | 4.8 | 0.08 | [31] |
| layered hydroxide | water | 0.7 | 1.3 | 16.1 | / | / | / | [32] |
| wood | water | 1.1 | 320 | 67.5 | 11 | 2.48 | 10.29 | [33] |
| ink | moisture | 1.1 | 3.6 | 130 | 13.89 | 0.98 | 1.6 | [34] |
| hydrogel | water | 0.21 | 95.35 | 262.5 | 140 | 0.71 | 0.48 | [35] |
| **This work （1 M K_2_CO_3_ )** | **1 M K_2_CO_3_** | **1.26** | **922.8** | **521.3** | **240** | **0.08** | **0.12** | **This work** |

**Supplementary References**

[1] a) P. A. Kralchevsky, K. Nagayamaa, *Adv. Colloid Interface Sci.* **2000**, *85*, 145; b) P. A. Kralchevskyt, K. Nagayama, *Langmuir* **1994**, *10*, 23.

[2] a) J. W. M. Bush, D. L. Hu, *Annu. Rev. Fluid Mech.* **2006**, *38*, 339; b) D. Liu, A. Mahmood, D. Weng, J. Wang, *Langmuir* **2019**, *35*, 16146; c) G. Lu, G. Zhu, Q. Zhang, P. Tian, M. Cheng, F. Shi, *Angew. Chem. Int. Ed.* **2023**, *62*, e202300448.

[3] L. Chen, X. Wei, Y. Sun, Y. Xue, J. Wang, Q. Wu, C. Ma, X. Yang, G. Duan, F. Wang, S. Jian, W. Yang, S. Jiang. *Chem. Eng. J.* **2022**, *446*, 137072.

[4] a) F. S. Marinkovic, D. M. Popovic, J. D. Jovanovic, B. S. Stankovic, B. K. Adnadjevic, *Appl. Phys. A-Mater.* **2019**, *125*, 611; b) F. Liu, Y. Liu, Y. Guo, J. Liu, J. Dong, T. Wang, D. Hao, Y. Zhang, *Carbohyd. Polym.* **2024**, *339*, 122229.

[5] a) Y. Fang, K. Wang, Q. Li, C. Huang, *Int. J. Pharmaceut.* **2021**, *607*, 121047; b) A. A. Martha, S. Sutarno, N. Nuryono, *Solid State Phenom.* **2022**, *339*, 11-17.

[6] M.-M. Iftime, I. Rosca, A.-I. Sandu, L. Marin, *Int. J. Biol. Macromol.* **2022**, *205*, 574.

[7] a) X. Wen, Z. Sun, X. Xie, Q. Zhou, H. Liu, L. Wang, X. Qin, S. C. Tan, *Adv. Funct. Mater.* **2023**, 2311128; b) F. Yu, J. Li, Y. Jiang, L. Wang, X. Yang, Y. Yang, X. Li, K. Jiang, W. Lü, X. Sun, *Adv. Sci.* **2023**, *10*, . 2302941

[8] Y. Koyano, N. J. Suematsu, H. Kitahata, *Phys. Rev. E* **2019**, *99*, 022211.

[9] Y. Koyano, M. Gryciuk, P. Skrobanska, M. Malecki, Y. Sumino, H. Kitahata, J. Gorecki, *Phys. Rev. E* **2017**, *96*, 012609.

[10] Y. Watahiki, T. Nomoto, L. Chiari, T. Toyota, M. Fujinami, *Langmuir* **2018**, *34*, 5487.

[11] M. Frenkel, G. Whyman, E. Shulzinger, A. Starostin, E. Bormashenko, *Appl. Phys. Lett.* **2017**, *110*, 131604.

[12] R. Seemann, J.-B. Fleury, C. C. Maass, *The Eur. Phys. J-Spec. Top.* **2016**, *225*, 2227.

[13] M. Li, M. Brinkmann, I. Pagonabarraga, R. Seemann, J.-B. Fleury, *Commun. Phys.* **2018**, *1*, 23.

[14] C. H. Ooi, A. van Nguyen, G. M. Evans, O. Gendelman, E. Bormashenko, N.-T. Nguyen, *RSC Adv.* **2015**, *5*, 101006.

[15] L. Zhang, Y. Yuan, X. Qiu, T. Zhang, Q. Chen, X. Huang, *Langmuir* **2017**, *33*, 12609.

[16] Y. Ikezoe, J. Fang, T. L. Wasik, T. Uemura, Y. Zheng, S. Kitagawa, H. Matsui, *Adv. Mater.* **2014**, *27*, 288.

[17] Y. Ikezoe, J. Fang, T. L. Wasik, M. Shi, T. Uemura, S. Kitagawa, H. Matsui, *Nano Lett.* **2015**, *15*, 4019.

[18] D. Li, F. Guo, Z. Cui, J. Zhou, Y. Zhai, Y. Du, J. Liu, N. Wang, Y. Zhao, *ACS Appl. Mater. Interfaces* **2020**, *12*, 53503.

[19] G. Zhao, T. H. Seah, M. Pumera, *Chem-Eur J.* **2011**, *17*, 12020.

[20] Y. Liang, H. Wang, D. Yao, Y. Chen, Y. Deng, C. Wang, *J. Mater. Chem. A* **2017**, *5*, 18442.

[21] K. Furukawa, T. Teshima, Y. Ueno, *Sci. Rep.* **2017**, *7*, 9323.

[22] C. C. Piras, D. K. Smith, *Chem-Eur J.* **2021**, *27*, 14527.

[23] Z. Mao, G. Shimamoto, S. Maeda, *Colloids Surf. A Physicochem. Eng. Asp.* **2021**, *608*, 125561.

[24] H. Wang, X. Gu, C. Wang, *ACS Appl. Mater. Interfaces* **2016**, *8*, 9413.

[25] T. Cai, L. Lan, B. Peng, C. Zhang, S. Dai, C. Zhang, J. Ping, Y. Ying, *Nano Lett.* **2022**, *22*, 6476.

[26] T. G. Yun, J. Bae, A. Rothschild, I.-D. Kim, *ACS Nano* **2019**, *13*, 12703.

[27] F. Gong, H. Li, Q. Zhou, M. Wang, W. Wang, Y. Lv, R. Xiao, D. V. Papavassiliou, *Nano Energy* **2020**, *74*, 104922.

[28] H. Cheng, Y. Huang, F. Zhao, C. Yang, P. Zhang, L. Jiang, G. Shi, L. Qu, *Energ. & Environ. Sci.* **2018**, *11*, 2839.

[29] J. Bae, T. G. Yun, B. L. Suh, J. Kim, I.-D. Kim, *Energ. & Environ. Sci.* **2020**, *13*, 527.

[30] T. Ding, K. Liu, J. Li, G. Xue, Q. Chen, L. Huang, B. Hu, J. Zhou, *Adv. Funct. Mater.* **2017**, *27*, 1700551.

[31] X. Huangfu, Y. Guo, S. M. Mugo, Q. Zhang, *Small* **2023**, *19*, 2207134.

[32] J. Sun, P. Li, J. Qu, X. Lu, Y. Xie, F. Gao, Y. Li, M. Gang, Q. Feng, H. Liang, X. Xia, C. Li, S. Xu, J. Bian, *Nano Energy* **2019**, *57*, 269.

[33] K. Zhang, X. Li, C. Yan, R. Shi, Z. Fang, S. Zhou, R. Cao, J. Tian, *ACS Nano* **2024**, *18*, 10259.

[34] T. He, H. Wang, B. Lu, T. Guang, C. Yang, Y. Huang, H. Cheng, L. Qu, *Joule* **2023**, *7*, 935.

[35] N. He, H. Wang, F. Li, B. Jiang, D. Tang, L. Li, *Energ. & Environ. Sci.* **2023**, *16*, 2494.
